# Spin‐Selective Interface Engineering in Oxide–Ferromagnetic Junctions via Atomic‐Scale Oxygen Control

**DOI:** 10.1002/advs.202523165

**Published:** 2026-02-20

**Authors:** David Maximilian Janas, Mira Sophie Arndt, Jonah Elias Nitschke, Lasse Sternemann, Valentin Mischke, Vitaliy Feyer, Iulia Cojocariu, Daniel Baranowski, Alessandro Sala, Andreas Windischbacher, Peter Puschnig, Jan Dreiser, Stefano Ponzoni, Giovanni Zamborlini, Mirko Cinchetti

**Affiliations:** ^1^ Department of Physics TU Dortmund University Dortmund Germany; ^2^ Peter Grünberg Institute (PGI‐6) Forschungszentrum Jülich GmbH Jülich Germany; ^3^ CNR – Istituto Officina Dei Materiali (IOM) Trieste Italy; ^4^ Institut Für Physik Karl‐Franzens‐Universität Graz NAWI Graz Graz Austria; ^5^ Swiss Light Source Paul Scherrer Institute (PSI) Villigen Switzerland

**Keywords:** epitaxial growth, magnetic tunneling junctions, MgO/Fe interface, momentum microscopy, spintronics

## Abstract

Atomic‐scale control of oxide–ferromagnet interfaces is crucial for optimizing spintronic heterostructures, yet interfacial oxygen remains difficult to control and verify. Here, we deterministically tune the prototypical MgO/Fe(100) interface from oxygen‐free terminations to fully intercalated oxygen layers by reactive growth under controlled O_2_ exposure, while preserving epitaxy. Momentum‐resolved photoemission identifies oxygen‐dependent fingerprints in *k*‐space that originate from the buried interface and persist up to a thickness of 8 layers of MgO. Insights from complementary spectroscopic methods link these *k*‐space signatures to interfacial chemistry, structural order, work‐function shifts, and an oxygen‐induced interface resonance within the MgO gap that alters the tunneling response. The combined results define a calibrated growth protocol that allows reproducibly preparing and identifying three distinct terminations — oxygen‐free, partially oxidized, and oxygen‐intercalated — and enables post‐growth conversion even in thicker films. Complementary spin‐resolved experiments reveal that oxygen‐free interfaces exhibit pronounced suppression of minority‐spin spectral weight at the Fermi level, consistent with coherent spin filtering across crystalline MgO, whereas oxygen intercalation reduces the spin contrast at *E*_F_. By turning interfacial oxygen from an uncontrolled variable into a measurable, adjustable parameter, our approach establishes MgO/Fe(100) as a benchmark platform for optimizing spintronic functionality in oxide/metal junctions.

## Graphical abstract

We show that interfacial oxygen at the MgO/Fe interface can be transformed from an uncontrolled variable into a tunable design parameter for spintronic junctions. Momentum microscopy provides direct access to the momentum‐ and spin‐resolved electronic structure beneath the insulating MgO layers, revealing how oxygen intercalation reshapes buried Fe states and governs spin‐dependent properties.

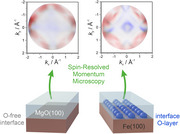

## 1 Introduction

Interfaces between oxides and ferromagnetic metals are foundational to spintronic technologies, underpinning both the long‐established tunneling magnetoresistance (TMR) effect and the more recently emerging concept of voltage‐controlled magnetic anisotropy (VCMA), which together enable high‐performance non‐volatile memories, magnetic sensors, and low‐power computing [1, 2, 3, 4, 5, 6, 7, 8]. In magnetic tunneling junctions (MTJs), TMR relies on coherent spin‐polarized tunneling across a thin insulating barrier, which acts as a spin‐dependent filter for the electron current flowing between two ferromagnetic electrodes. In this context, crystalline MgO has emerged as the benchmark material due to its ability to support highly efficient symmetry‐filtered transport at ferromagnet/MgO interfaces [9], far surpassing earlier Ge‐ or Al_2_O_3_‐based junctions [10, 11]. In Fe/MgO/Fe MTJs, theory predicted TMR ratios beyond 1000% [12], and, when electronic correlations are included, even beyond 4000% [13]. Yet, despite remarkable advances — from TMR values of 200% in early reports [2, 14] to 417% at room temperature and 914% at 3 K [15] — experiments still fall short. Unintended oxidation of Fe during MgO deposition has been identified as a principal contributor to this discrepancy [16, 17, 18, 19].

However, the role of oxygen at Fe/MgO interfaces is intrinsically dual. On the detrimental side, the oxygen at the interface hybridizes with Fe 3*d* states and reshapes the interfacial DOS near the Fermi level, weakening symmetry‐selective spin filtering and lowering the spin polarization at *E*_F_, thereby limiting TMR [16, 17, 20, 21, 22]. In addition, excess oxygen degrades the interfacial perpendicular magnetic anisotropy (iPMA) [8, 23, 24] essential for scalable, thermally stable devices: both over‐ and under‐oxidation introduce disorder and disrupt the O *p_z_* and Fe $d_{z^{2}}$ overlap, thereby diminishing iPMA [8, 23, 24]. On the beneficial side, engineered oxidized Fe layers at the interface can enhance VCMA by amplifying electric‐field‐driven changes in orbital occupation and magnetocrystalline anisotropy [25, 26]. Moreover, exchange‐bias effects, where antiferromagnetic FeO patches at the interface pin the magnetization of the Fe layer, have also been observed and enhanced by increasing the interface oxygen content [27]. Other first‐principles calculations even suggest that, under specific bonding configurations, interfacial oxygen could enhance spin polarization at the Fermi level [28].

The key challenge thus lies in controlling the amount of oxygen at the buried interface and understanding its role in determining the properties of the interface. Here, we achieve atomic‐scale control in MgO/Fe(100) junctions, spanning from atomically sharp, oxygen‐free terminations to a complete interfacial oxygen layer, all while preserving crystalline quality. However, probing such buried configurations by photoemission is intrinsically difficult: the photon energies typically used for valence‐band spectroscopy result in a short electron mean free path that conceals the spectroscopic fingerprints of Fe [29]. To overcome this limitation, we employ momentum‐resolved photoemission, i.e. momentum microscopy (MM), which captures characteristic momentum patterns of Fe even beneath MgO films up to 8 monolayers (MLs) (≈  1.7 nm, using 1 ML = 0.21 nm for MgO(100) interlayer distance), where conventional spectra become indistinct. In this way, MM uncovers pronounced oxygen‐dependent variations near the Fermi level, while a distinct resonance around −1.8 eV emerges as an additional spectroscopic signature of interfacial oxygen incorporation.

We further apply spin‐resolved MM (spin‐MM) to access, with momentum resolution, the spin character of interfacial states beneath insulating overlayers — an experimental capability long sought but not realized until now. With this tool, we can directly visualize how interfacial oxygen reshapes spin polarization and, conversely, identify the pristine fingerprint of spin‐selective tunneling in its absence.

The MM and spin‐MM data are supported by a comprehensive suite of surface‐sensitive techniques, including low‐ and medium‐energy electron diffraction (LEED and MEED), X‐ray photoelectron and absorption spectroscopies (XPS and XAS), Auger electron spectroscopy (AES), and scanning tunneling microscopy and spectroscopy (STM and STS). Altogether, this combined synthesis–characterization approach yields a calibrated growth‐and‐readout protocol that prepares and verifies three distinct interface terminations: oxygen‐free, partially oxidized, and oxygen‐intercalated. Beyond MgO/Fe, this approach generalizes to buried oxide/metal junctions, turning interfacial oxygen from an uncontrolled variable into a practical design parameter for device‐relevant interfaces in spintronic applications.

## 2 Results

Uncontrolled oxidation is a well‐known challenge in MgO/Fe growth. Conventional deposition routes — such as reactive Mg evaporation in O_2_ atmosphere [18, 30, 31] or electron‐beam evaporation of MgO [19, 32, 33] — frequently lead to formation of a sub‐stoichiometric FeO layer at the interface. Proposed mechanisms include residual gas adsorption, catalytic O_2_ dissociation by Mg atoms [18], or direct oxidation of Fe at elevated temperatures [34, 35, 36]. To stabilize film growth and improve reproducibility, it has been suggested to intentionally passivate the Fe surface, prior to MgO deposition, by creating a controlled Fe–O layer [32, 37, 38].

Building on this concept, we adopt a dual approach: tuning the O_2_ background pressure during Mg deposition while simultaneously exploiting templates with opposite characteristics, from an oxygen‐free (pristine Fe(100)) to a pre‐formed and well‐ordered oxygen overlayer (oxygen‐passivated Fe(100)‐*p*(1 × 1)O) surface template, hereafter referred to as **Ref‐Fe** and **Ref‐FeO**, respectively. Note that the latter does not represent bulk FeO, but rather a single layer of oxygen atoms occupying hollow sites atop Fe(100), sometimes termed Fe–O in the literature. By adjusting the oxygen pressure between 3 × 10^−9^ and 7 × 10^−8^ mbar, we access a continuum of interface configurations, ranging from oxygen‐free MgO/Fe terminations to interfaces containing a complete interfacial O layer. The latter structurally corresponds to the passivated Fe–O surface, but remains buried beneath the MgO film. For clarity, we distinguish three representative cases: oxygen‐free MgO/Fe (**MgO‐fO**), interfaces with partial interlayer oxygen (**MgO‐pO**), and MgO grown on a complete interfacial O layer (**MgO‐iO**).

All films were deposited at 440 K, as this temperature optimizes surface morphology [31]. MEED oscillations confirmed a well‐ordered, layer‐by‐layer growth mode on both substrates, with more pronounced oscillations on Ref‐FeO, confirming the established enhanced crystallinity during the early stages of MgO deposition on this surface [16, 39, 40]. Details are provided in Section S1. Unless stated otherwise, the data presented in this work were acquired on films subjected to a standardized post‐deposition anneal to approximately 870 K (without further O_2_ supply), which reproducibly yielded the highest crystalline quality and the most well‐defined spectroscopic response in our preparation protocol. In addition, selected films were annealed to other temperatures to assess the thermal stability of the MgO/Fe interface and to track the evolution of interfacial oxidation upon thermal treatment.

### 2.1 The Presence of Interfacial Oxygen in Photoelectron Spectroscopy

**FIGURE 1:**
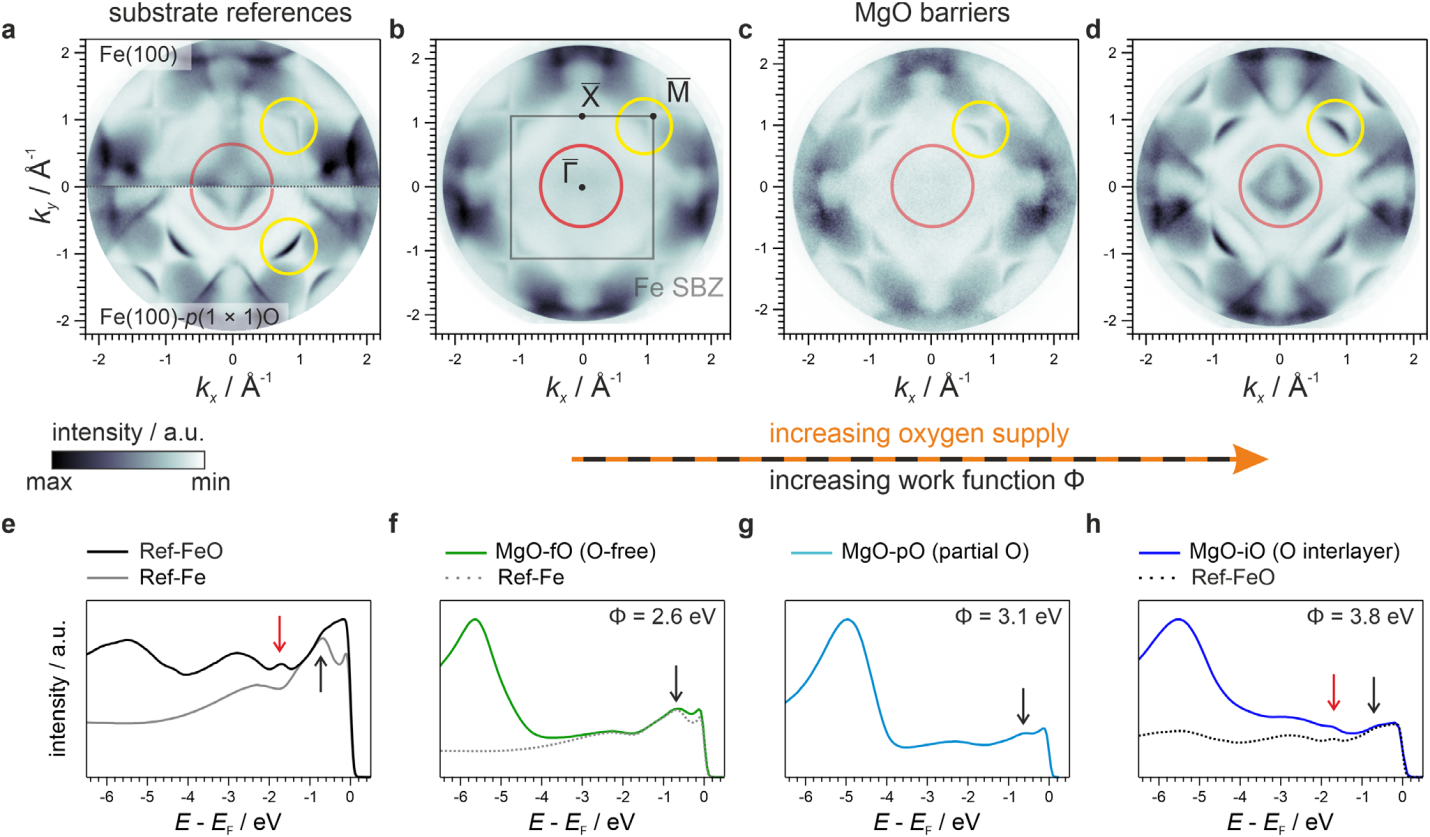
Evolution of the interfacial electronic structure during MgO growth on Fe(100) revealed by photoelectron spectroscopy. a) Momentum‐resolved photoemission maps at the Fermi energy for Ref‐Fe (top) and Ref‐FeO (bottom). The corresponding work functions are 4.0 eV for Ref‐Fe and 4.4 eV for Ref‐FeO. Yellow circles highlight features that change significantly due to the presence of interfacial oxygen. b)‐d) Fermi maps for 2 ML MgO films grown on Fe(100) under varying O_2_ back pressures: b) p_b_ = 5.0 × 10^−9^ mbar, c) p_c_ = 1.5 × 10^−8^ mbar, and d) p_d_ = 4.0 × 10^−8^ mbar. The corresponding work functions of the MgO/Fe systems increase progressively, reflecting the impact of excess oxygen at the interface. e)‐h) Normalized momentum‐integrated energy distribution curves (EDCs) for the same systems presented above. Black and red arrows indicate the positions of spectral features associated with the absence and presence of a full interfacial oxygen layer, respectively. All measurements were performed using p‐polarized light with a photon energy of h υ=64 eV. Data for the Ref‐Fe and Ref‐FeO substrates in panel a) are adapted from Ref. [42] to serve as a reference baseline.

Panel (a) of Figure 1 compares photoemission momentum maps recorded at the Fermi energy (*E—E*_F_ = 0 eV) for Ref‐Fe and Ref‐FeO, the two substrate templates used in this study. Oxygen passivation in Ref‐FeO produces a clear momentum‐space fingerprint: additional intensity appears in the outer regions of the maps (yellow circles), consistent with hybridization between O and Fe states [41, 42]. In contrast, the central spectral weight (red circle) shows comparable intensity for both substrates, highlighting a part of the electronic structure that remains largely unaffected by passivation. These signatures provide a reference against which the effect of MgO deposition under different growth conditions can be evaluated. For clarity, the corresponding first surface Brillouin zone (SBZ) of Fe(100) and the corresponding high symmetry points are sketched in Figure 1b.

With this baseline established, we next follow how the characteristic features in the momentum maps evolve under gradually increasing O_2_ pressure during Mg deposition (Figure 1b–d). Although the Fe‐derived photoemission intensity is progressively attenuated with increasing MgO coverage, distinct substrate features remain clearly visible in the momentum maps. This persistence highlights the electronic transparency of MgO around the Fermi level: despite the insulating overlayer, *k*‐space fingerprints of the buried interface remain extremely sharp. The effect is facilitated by the epitaxial match and high crystalline quality of the MgO films. In the outer regions of the maps, the established oxygen‐sensitive features evolve systematically and gradually approach the electronic signatures of Ref‐FeO. We therefore conclude that this continuous evolution provides unambiguous photoemission fingerprints of the degree of interfacial passivation and demonstrates that, at sufficiently high O_2_ exposure, oxygen becomes intercalated at the interface.

Instead, at lower O_2_ exposures (Figure 1b,c), the momentum maps reveal a pronounced suppression of intensity in the central regions. This effect depends strongly on the incident light polarization (see Figure S3 in Section S2): while the central intensity is suppressed under *p*‐polarization, it largely reappears when switching to *s*‐polarization. This polarization dependence indicates that the missing intensity under *p*‐polarization originates from pronounced linear dichroism in the occupied electronic states, pointing to a strong anisotropic orbital character of the interfacial states at oxygen‐free MgO‐fO interfaces. Notably, enhanced linear dichroism is likewise observed for the unoccupied states probed by XAS at the Mg and O edges, providing complementary evidence for pronounced orbital polarization at these atomically sharp, MgO‐fO terminations (see Figure S4 in Section S2). Together, our findings demonstrate that the presence or absence of interfacial oxygen directly influences not only the electronic structure but also the symmetry and polarization characteristics of interfacial states [23].

**FIGURE 2:**
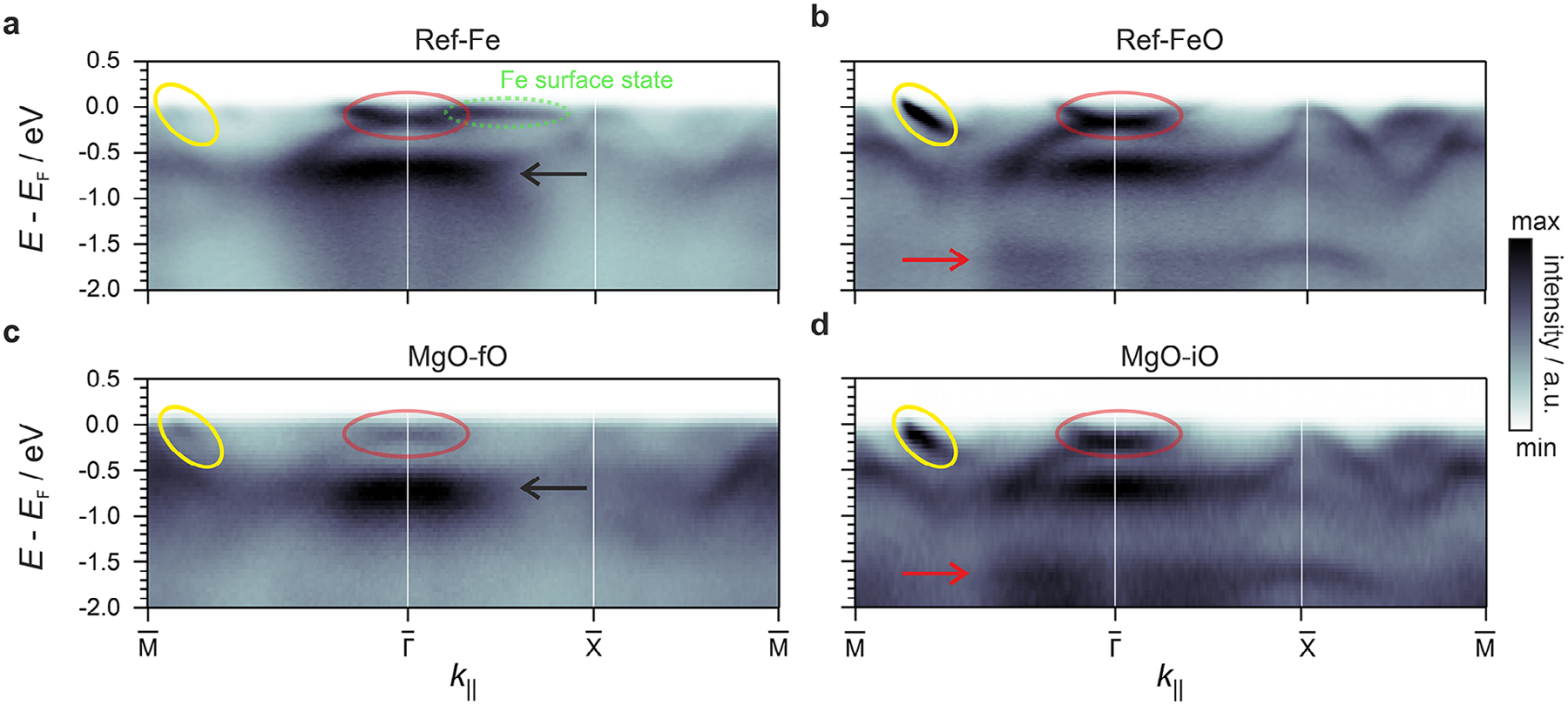
Band‐structure cuts of the MM intensity along the high‐symmetry directions of the Fe(100) SBZ, focusing on an energy window around E_F_ dominated by Fe d states. The band dispersions of the states marked in Figure 1 are highlighted accordingly by red and yellow circles, and the corresponding peak positions in the related EDCs are indicated by red and black arrows, respectively. In addition, a prominent Fe surface state along the $\bar{\Gamma X}$−direction is marked in green. Panels show a) Ref‐Fe, b) Ref‐FeO, c) MgO‐fO, and d) MgO‐iO. The Ref‐Fe and Ref‐FeO results are based on data from Ref. [42], replotted here for direct comparison with the MgO interfaces.

Momentum‐integrated energy distribution curves (EDCs) in panels (e–h), obtained by summing the *k*‐maps over the full momentum range (*k*_x_, *k*_y_ ∈[−2.0,2.0] Å^−1^), confirm the interfacial oxidation and provide additional spectroscopic fingerprints of the different interface terminations. At low O_2_ exposure, the EDCs exhibit a pronounced feature at –0.8 eV (black arrows), characteristic of oxygen‐free (MgO‐fO) interfaces, which progressively diminishes and merges into a plateau‐like region near the Fermi level as the O_2_ pressure increases. By contrast, a peak at −1.8 eV (red arrows), attributed to an oxygen‐induced surface resonance [43], signals the formation of fully intercalated oxygen layers (MgO‐iO). The momentum‐resolved dispersive character of the identified spectral features is illustrated in Figure 2, which displays band‐structure cuts extracted from the dataset shown in Figure 1 and includes the same Ref‐Fe and Ref‐FeO dispersions discussed in Ref [41]. Oxygen chemisorption on Fe(100) was shown there to enhance electronic correlations in the topmost Fe layer, leading to pronounced modifications close to *E*_F_ (notably a narrowing of the Fe *d*‐band density of states and a reduced exchange splitting) that crucially influence its surface chemical reactivity [41, 44]. Moreover, electron correlation drives dynamic spin filtering in Ref‐FeO, manifesting as a pronounced spin‐dependent broadening of the O‐derived bands at higher binding energies [41]. Importantly, we showed that these correlation‐driven changes are not captured quantitatively by conventional single‐particle approaches, whereas correlation‐aware methods such as DFT+DMFT reproduce the experimentally observed band features with high accuracy [41]. The yellow and red ellipses mark the states highlighted in the Fermi maps and trace their evolution along the high‐symmetry directions of the Fe(100) SBZ. To enhance visual comparability, all intensities were normalized to the feature marked by the black arrow at –0.8 eV.

This comparison demonstrates that the oxygen‐free interfaces — Ref‐Fe (Figure 2a) and MgO‐fO (Figure 2c) — display similar dispersions, yet with pronounced intensity variations. On Ref‐Fe, the well‐established [45, 46, 47] Fe minority surface state (green) is observed along the $\bar{\Gamma X}$−direction, which vanishes upon MgO deposition. In addition, the feature around the $\bar{\Gamma }$‐point (red circle) becomes significantly attenuated compared with the state at –0.8 eV mentioned earlier (black arrow), indicating a strong suppression of this entire dispersive branch upon MgO coverage.

In contrast, the oxygen‐terminated interfaces, Ref‐FeO (Figure 2b) and MgO‐iO (Figure 2d), display seemingly identical dispersions, including an oxygen‐induced band near –1.8 eV (red arrow) that corresponds to the reported O resonance. This close correspondence demonstrates that the interfacial oxygen layer governs the electronic properties of the topmost Fe surface layer, effectively overriding differences in the overlayer structure. The broad features emerging between –5.0 and –6.0 eV in the valence band are attributed to oxygen states within the MgO film [19, 48]. To visualize these higher‐energy states and disentangle MgO‐ and FeO‐derived contributions, additional band‐structure cuts obtained with *s*‐polarized light are provided in Figure S5 (Section S3), where the reduced number of visible bands enables a clear assignment. Moreover, symmetry‐equivalent cuts along orthogonal directions are compared to O 2*p*‐projected slab density functional theory (DFT) of the topmost MgO layer to identify and highlight their orbital character across the employed light polarizations (Figure S6 and Section S3).

**FIGURE 3:**
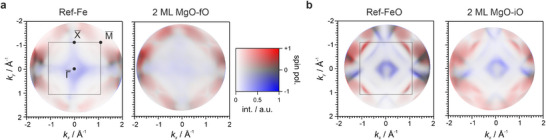
a) Spin‐resolved Fermi maps (*E* = *E*_F_) for Ref‐Fe (left) and the corresponding MgO‐covered interface without interfacial oxygen 2 ML MgO‐fO (right). The spectral weight in the minority spin channel is strongly attenuated upon MgO deposition, while the majority states remain prominent, consistent with spin‐selective tunneling across the crystalline MgO barrier. b) Fermi maps for oxygen‐passivated Ref‐FeO (left) and the fully intercalated MgO‐iO interface (right). In contrast to a), both spin channels retain comparable intensity, indicating loss of spin contrast due to oxygen‐induced hybridization. All maps were recorded using p‐polarized light at 64 eV. The 2D color‐scale encodes both spectral intensity and spin contrast. The data highlight how interfacial oxygen modulates the probed spin‐dependent electronic structure at the buried interface. Note that the Ref‐Fe and Ref‐FeO maps correspond to data previously analyzed in Ref. [42].

Since spintronic performance is ultimately governed by the spin‐dependent electronic structure of the buried interfaces, we employed spin‐MM to further investigate 2 ML MgO films grown on both Ref‐Fe and Ref‐FeO templates (Figure 3). At this thickness, charge transport already proceeds via tunneling, as in thicker device‑relevant barriers [9], while the reduced MgO thickness still provides sufficient photoemission intensity for spin analysis.

Figure 3a compares the spin‐resolved Fermi surface maps for the clean Ref‐Fe substrate and its oxygen‐free MgO‐covered counterpart (MgO‐fO). The Ref‐Fe surface shows clear spin contrast across the SBZ, as expected from its strong spin polarization. Upon deposition of MgO, the majority features remain clearly discernible, whereas the minority spectral weight is markedly attenuated — most notably around the Brillouin zone center — consistent with the spin‐filtering character of crystalline MgO barriers. While disorder may reduce overall sharpness and contrast, it is expected to act largely spin‐independently, and thus cannot explain the pronounced suppression of only the minority channel. Interface‐specific electronic‐structure modifications may also influence the apparent magnitude of the effect, but within the present data they cannot be separated from symmetry‐selective tunneling through MgO. Overall, the observed trend is consistent with the established spin‐filtering mechanism.

In contrast, Figure 3b presents the corresponding maps for oxygen‐passivated Ref‐FeO surface and for a 2 ML MgO film grown on top (MgO‐iO). Similar to pristine Ref‐Fe, both Ref‐FeO and MgO‐iO retain a strongly spin‐polarized electronic structure. However, unlike in MgO‐fO, both spin channels appear with comparable intensities, as the coexistence of red and blue features reflects contributions from majority and minority states. The residual non‐zero intensity in the minority channel at these oxygen‐rich interfaces suggests that interfacial oxygen alters the spin‐dependent spectral weight at the Fermi level, possibly by enabling additional hybridization pathways or by modifying the interfacial symmetry of electronic states. While a full theoretical analysis will be required for quantitative interpretation, the data clearly reveal a qualitative trend: oxygen‐free interfaces selectively suppress minority spin states, whereas oxygen‐intercalated terminations restore spin degeneracy at the Fermi level. This direct momentum‐resolved signature provides a sensitive probe of interface quality and spin selectivity, demonstrating that spin‐resolved momentum microscopy can provide spectroscopic hallmarks of buried spin‐filtering interfaces.

To substantiate the momentum‐resolved findings, we consult XPS measurements of the Fe 3*p* and Mg 2*p* core levels, which provide direct insight into the chemical environment at the MgO/Fe(100) interface (see Section S4). Representative spectra of MgO thin films (2–3 ML) are shown in Figure S7: for low oxygen content, the data reveal signatures of purely metallic Fe, whereas samples with high interfacial oxidation exhibit additional features associated with Fe^2+^ and Fe^3+^ species. Analysis of the O core levels further supports the formation of an intercalated oxygen layer, reflected in the strongly enhanced relative oxygen signal. Detailed fitting procedures and full spectral decompositions are presented in Section S4.

The evolution of the work function provides an additional macroscopic probe of interfacial oxidation. From the secondary electron cutoff in photoemission spectra, we find a systematic increase from Φ_b_ = 2.6 eV (MgO‐fO) to  Φ_c_ = 3.1 eV (MgO‐pO) and  Φ_d_ = 3.8 eV (MgO‐iO). This trend can be rationalized either by oxygen incorporated within the MgO film itself [49], or by excess oxygen directly located at the interface [50]. DFT calculations reproduce the experimental work function increase, yielding 2.2 eV for a 2 ML MgO‐fO interface and 3.2 eV for its 2 ML MgO‐iO counterpart, thereby underscoring the role of interfacial oxygen species in governing the work function of MgO/Fe systems.

### 2.2 Different MgO Growth Modes and Surface Quality Assessment

**FIGURE 4:**
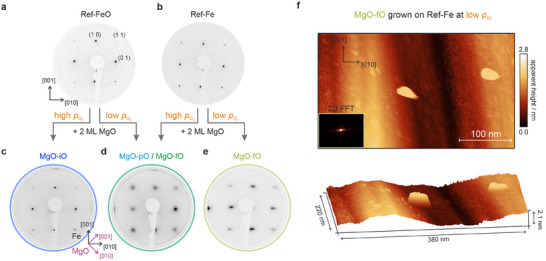
LEED and STM images illustrating different stages of sample preparation and MgO growth. The top row shows LEED images of a) an oxygen‐passivated Fe(100)‐p(1 × 1)O surface and b) a pristine Fe(100) film. The high‐symmetry directions of Fe(100) are indicated by the black arrows. The bottom row presents LEED images of 2 ML MgO thin films grown under varying conditions: c) a 2 ML MgO‐iO film grown on Ref‐FeO under high O_2_ pressure, d) typical MgO‐pO and MgO‐fO films grown either at low O_2_ pressure on Ref‐FeO, or at high O_2_ pressure on a Ref‐Fe substrate, and e) an MgO‐fO film grown under low O_2_ back pressure on top of clean Ref‐Fe. All LEED images were recorded at a kinetic energy of 90 eV. f) Topographic STM image of the MgO‐fO sample grown at low O_2_ pressure on Ref‐Fe (V_bias_ = +3.0 V, I = 100 pA). The surface exhibits a pronounced wavy pattern along the [010] direction, corresponding to the direction in which the LEED spots from Figure 4e are elongated. This corrugation is attributed to a warped MgO surface morphology. The inset in the top panel shows the 2D FFT of the measured topographic image.

Having established the oxidation regimes, we next employ LEED and STM to determine how the oxygen content influences the structural arrangement of the MgO films. As in the previous section, we first analyze the reference substrates to be used as a benchmark. Both Ref‐FeO and Ref‐Fe exhibit sharp LEED spots indicative of their high crystalline quality (Figure 4a,b, respectively). In contrast, bulk MgO samples show broader diffraction spots (see Figure S10c in Section S5) and even a complete disappearance of the pattern at primary energies below 35 eV. Such behavior is consistent with strong charging effects in MgO, in line with early secondary‐electron emission studies reporting a crossover from negative to positive charging around 33 eV [51]. This, and the recovery of sharp spots upon Fe deposition indicates that the broad features observed on actual bulk MgO are not due to poor crystalline quality, but are more plausibly linked to charging and the associated surface potentials.

Next, we examine the hybrid interfaces. Most notably, systems containing a fully intercalated oxygen layer (MgO‐iO) exhibit exceptionally sharp LEED spots (Figure 4c), comparable to those of the pristine substrates. This suggests the formation of well‐ordered MgO films. To grow such films, we obtained the best results by depositing Mg at high oxygen pressure on the pre‐passivated Ref‐FeO surface, followed by high annealing (≥ 870 K).

In contrast, films with only partial oxidation of the interface (MgO‐pO) produce more diffuse LEED patterns (Figure 4d), comparable to the ones of bulk MgO. Interestingly, such films can be obtained in two ways: by deposition at low oxygen pressure on Ref‐FeO, or by growing on Ref‐Fe under higher oxygen pressure and post‐deposition flash annealing at 870 K. Both approaches result in comparable structural quality, although passivation may promote a more uniform initial film morphology that does not require annealing, as shown in Figure S10 (Section S5). MgO growth starting from the Ref‐FeO substrate shows that the passivating oxygen layer is not stable during deposition but acts as an additional reservoir of oxygen atoms, directly affecting the oxygen stoichiometry within the dielectric film. To support this hypothesis, we prepared MgO films on both Ref‐FeO and Ref‐Fe under similar conditions. Quantifying the oxygen content using our MM analysis, we find an increase of about 0.3 eV in the work function for films on Ref‐FeO compared to Ref‐Fe (see Section S5 and Figure S11), consistent with enhanced oxygen incorporation.

Lastly, we observe a distinct behavior for MgO‐fO interfaces, obtained by growing MgO at our lowest O_2_ pressure directly on pristine Ref‐Fe. In LEED (Figure 4e), these low‐oxygen‐growth samples display elongated diffraction spots along the Fe [010] direction, indicating a pronounced anisotropy not observed under other growth conditions. To connect this reciprocal‐space signature to the real‐space morphology, we performed STM measurements (Figure 4f). The topography reveals a unidirectional corrugation along the [010] direction, reminiscent of a weakly warped or locally tilted surface associated with strain relaxation. From this modulation we estimate a surface tilt angle below 1°, significantly smaller than the misfit‐dislocation‐induced warping angles (∼3.5°) reported for thicker MgO films, where pseudomorphic growth breaks down [39]. Importantly, the 2D fast Fourier transform (FFT) of the STM image exhibits an anisotropic intensity distribution with an elongation that mirrors the LEED pattern, corroborating that the spot broadening originates from the same unidirectional modulation observed locally by STM. In addition, the surface is laterally heterogeneous: comparatively smooth regions alternate with granular patches (see Figure S12 and Section S6), pointing to locally increased disorder under limited oxygen availability (consistent with reports that insufficient oxygen supply may reduce structural order and, upon annealing, promote Mg segregation) [31].

Despite the morphological variations observed in the MgO‐fO regime, as evidenced by LEED and STM, MM analysis reveals that the MgO bands remain essentially unchanged and sharp, as illustrated in Figure S13 (Section S6). This finding indicates that the intrinsic electronic structure of the MgO film is largely unaffected by these deviations in surface morphology. The robustness of the band dispersion characteristic momentum space features is consistent with previous tunneling spectroscopy results, which likewise report stable MgO electronic states across varying surface conditions [52]. These results demonstrate that high‐quality MgO films can be grown on Fe(100) over a broad range of O_2_ pressures, encompassing interface terminations from MgO‐iO to MgO‐fO.

### 2.3 Chemical Composition and Stability of MgO/Fe Interfaces Under Varying Annealing Conditions

Beyond growth parameters, post‐deposition annealing is a critical lever for controlling stoichiometry and interfacial properties. We therefore systematically explored the interface evolution under thermal treatment using Auger electron spectroscopy (AES) in addition to our MM approach.

**FIGURE 5:**
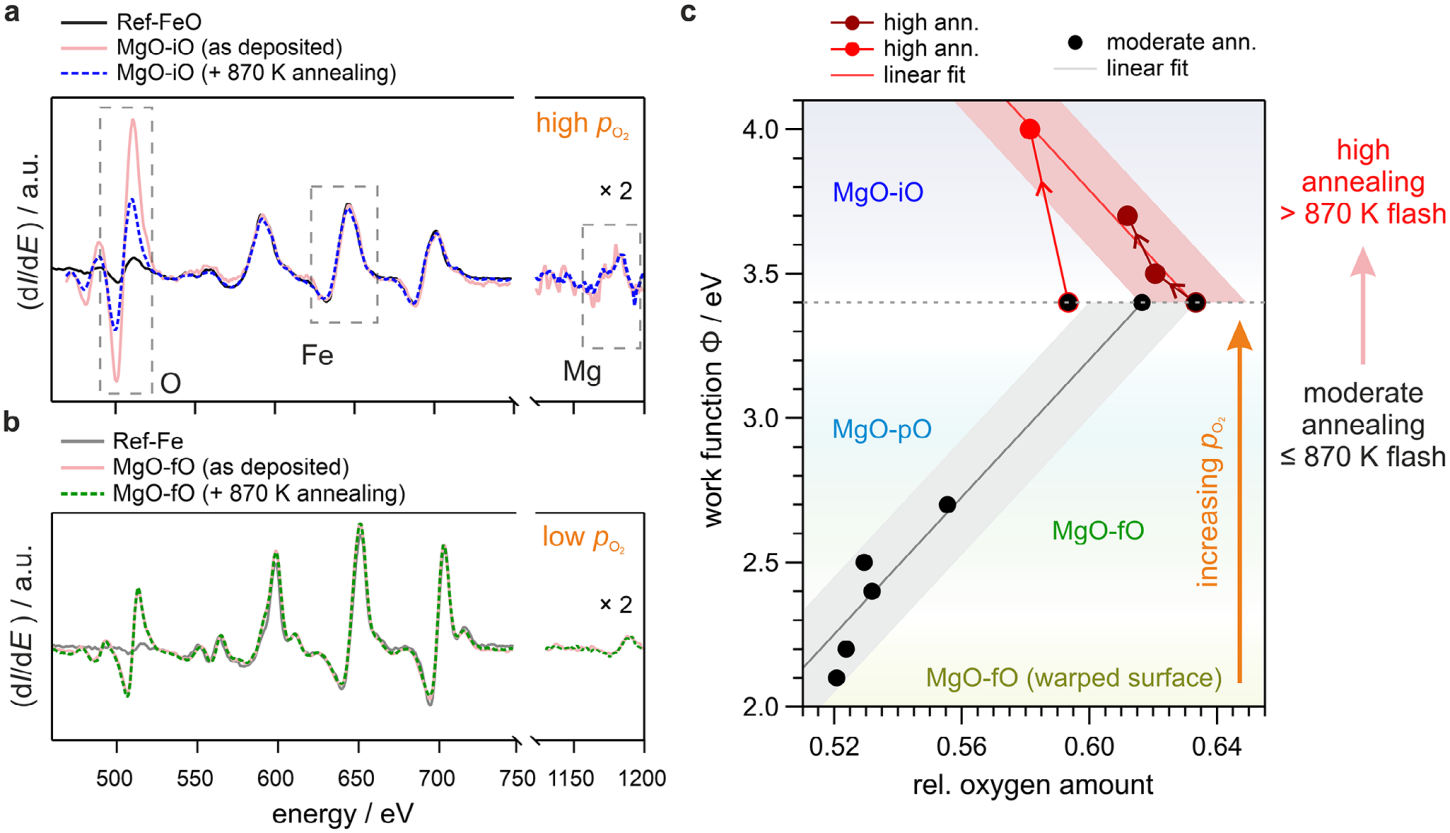
AES and work function analysis of MgO/Fe interfaces under varying growth and annealing conditions. a) AES spectra for Ref‐FeO (black curve) and an MgO‐iO film grown at high O_2_​ back pressure, shown right after deposition (light red) and after flash annealing at 870 K (blue). The preparation of samples under high O_2_ back pressure is necessary to form the interfacial oxygen layer. b) AES spectra for clean Ref‐Fe and the resulting MgO‐fO system right after growth (light red), and after annealing at 870 K (dotted green curve). The film was grown under low O_2_ pressure. For clarity, the intensity in the region of the Mg peak is scaled by a factor of 2 across all presented spectra. c) Work functions of differently prepared thin MgO/Fe interfaces plotted against the relative oxygen amount (with respect to the Mg amount). The chemical composition was extracted from the peak‐to‐peak intensity ratios of the Auger spectra. Data points above the dashed grey line correspond to samples annealed at temperatures exceeding 870 K, while points below the line represent samples flash annealed up to 870 K. Shaded grey and red regions highlight the estimated work function uncertainties. Arrowheads along connecting lines indicate stepwise annealing sequences.

AES allows direct measurement of the peak‐to‐peak intensities of O, Fe, and Mg signals, which we use to estimate the relative contributions of oxygen and magnesium. Figure 5a shows AES spectra for a Ref‐FeO sample (black curve) and an MgO‐iO film deposited under high O_2_ back pressure, measured immediately after deposition (light red curve) and after flash annealing at 870 K (blue dotted curve). The dashed grey boxes highlight the relevant elemental peaks: oxygen at around 500 eV, iron at 650 eV, and magnesium near 1200 eV, which were used for chemical analysis of the samples. To improve visibility, the signal from 1100 eV onward has been magnified by a factor of two. The pronounced reduction of the oxygen peak upon annealing, while the magnesium peak remains essentially unchanged, indicates that excess oxygen is removed without compromising the MgO layer. However, additional annealing beyond 870 K results in a concurrent decrease of both the oxygen and magnesium signals, indicating the onset of thermal degradation of the MgO‐iO film (see Section S8 and Figure S15).

For comparison, Figure 5b shows spectra of a clean Ref‐Fe surface (grey) and an MgO‐fO film grown at low O_2_ pressure, without signs of interfacial oxygen (light red), together with a spectrum acquired after annealing the film at 870 K (dotted green). The MgO‐fO spectra remain unchanged even after annealing, demonstrating a chemically stable interface and confirming the absence of interfacial O under these growth conditions, where no excess O is expected.

To investigate these trends further, Figure 5c plots work functions against the oxygen amount relative to magnesium, derived from weighted peak‐to‐peak intensity ratios in AES spectra (details of the estimation are illustrated in Section S7). Additionally, we sketch our classification of oxygen passivation according to the work function. Two distinct regions can be observed and linked to the annealing temperature. For low oxygen content (MgO‐fO and MgO‐pO), moderate annealing below 870 K is advisable, as it improves the ordering at the interface and leads to a significant sharpening of both substrate and overlayer spectral features (see Figure S16 in Section S8). In this regime, the work function increases almost linearly with increasing amount of oxygen. However, when approaching the almost fully passivated interface at a threshold of about 3.4 eV, this trend reverses, and the work function increases only as oxygen content decreases. This behavior is observed upon annealing at higher temperatures (T > 870 K) and reflects the aforementioned instability of the MgO‐iO film at elevated temperatures. Interestingly, complementary MM data in Section S8 (Figure S15) show sharpening of the spectral features associated with intercalated oxygen layers, suggesting that the ordering near the interface improves even as the overall MgO film integrity decreases.

It is worth noting that while thin MgO films grown on Ref‐Fe exhibit a slightly oxygen‐rich composition, control measurements on thicker films (> 8 ML), typically deposited at higher O_2_ partial pressures, show only minor deviations from the ideal relative oxygen amount of 0.5 corresponding to a Mg:O = 1:1 ratio. In the next section, we extend our study to these thicker MgO films, where strain relaxation sets in and the growth evolves beyond the pseudomorphic limit. This provides an opportunity to assess how increasing film thickness impacts structural and electronic properties, and to clarify whether interfacial oxygen remains a decisive factor in this regime.

### 2.4 Beyond the Pseudomorphic MgO Growth: Thick MgO Barriers

Thick MgO films grown on Ref‐Fe exhibit structural features that differ significantly from their ultrathin counterparts due to strain relaxation effects. While MgO grows pseudomorphically up to approximately 6 ML, the lattice mismatch between MgO and Fe (3.5%) leads to strain release beyond this thickness, resulting in the formation of misfit dislocations [16, 39]. These structural transitions are clearly visible in LEED patterns as satellite spots surrounding the main diffraction peaks — a cross‐shaped pattern characteristic of thick MgO layers.

**FIGURE 6:**
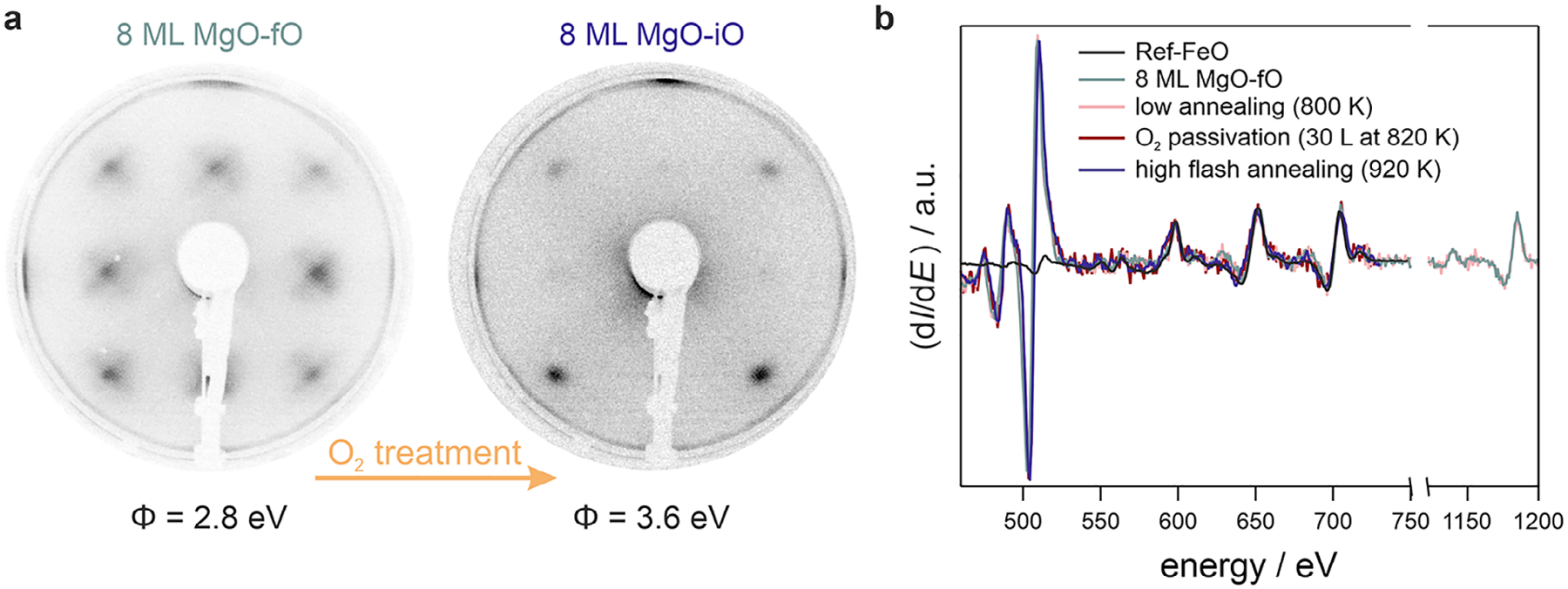
LEED images and Auger spectra of an 8 ML MgO film grown on Ref‐Fe. a) LEED diffraction patterns of the MgO film after growth and gentle post‐deposition annealing to 800 K (left), and subsequent oxygen treatment, which includes oxygen exposure at elevated temperatures, followed by high‐temperature annealing (right). The oxygen treatment increases the work function from 2.8 eV to 3.6 eV. b) Auger spectra recorded during the different preparation steps: starting with the clean substrate, followed by MgO growth, post‐deposition annealing, oxygen treatment, and high‐temperature annealing. Notably, no evident change in the chemical composition is observed during the treatment of the thick MgO film, while MM data indicate a transition from an MgO‐fO to an MgO‐iO interface.

The left panel in Figure 6a shows the LEED pattern of a thick (∼8 ML) MgO‐fO interface, highlighting these satellite spots alongside slightly diffuse primary diffraction peaks. The work function measured for this sample is Φ  =  2.8 eV, which lies in the range of values for MgO‐pO thin films (2–3 ML), while showing only minimal interfacial contributions in comparison to these thinner films.

To investigate whether oxygen intercalation can modify such stabilized thick films, we subjected them to an oxygen treatment consisting of the standard passivation procedure to obtain MgO‐iO followed by annealing at T = 920 K. This process induces significant changes in the LEED pattern (Figure 6a, right): satellite spots disappear entirely, while (1 0) reflections become faintly visible and (1 1) reflections sharpen noticeably. Concomitantly, the work function increases substantially from Φ = 2.8 eV to Φ = 3.6 eV, indicating electronic modifications at or near the interface. In contrast to thin film behavior, AES measurements reveal no detectable differences in chemical composition before and after oxygen treatment (see Figure 6b). This stability suggests that while the thick MgO film remains chemically intact during annealing, the changes occur at the buried interface — likely linked to electronic structure rearrangements.

Our findings are complemented by MM data analysis presented in Figure S17 (Section S9): following oxygen treatment, new features emerge in the Fermi maps resembling those observed for thin films with intercalated oxygen layers. The persistence of MgO‐derived bands together with a severely attenuated Fe Fermi edge indicates that the films are continuous and chemically stable, making dewetting or film disruption unlikely. These observations therefore suggest that even beyond its pseudomorphic growth regime, thick MgO remains amenable to subsequent oxygen incorporation under appropriate conditions.

This demonstrates that it is not only possible to modify thin MgO films in a controlled fashion but also to convert thick MgO‐fO to MgO‐iO. Unlike thin films, thick MgO films appear structurally stable once they transition beyond pseudomorphic growth (> 6 ML), irrespective of the interface composition, which makes them an attractive target for post‐growth treatments.

### 2.5 Influence of Intercalated Oxygen on the Electronic Properties of the MgO/Fe Interface

Finally, the electronic structure of MgO/Fe(100) interfaces is investigated by combining energy distribution curves (EDCs) from MM measurements with scanning tunneling spectroscopy (STS) data. EDCs recorded for thick (8 ML) MgO films are systematically compared with those of ultrathin (2–3 ML) films, while the photoemission results in the ultrathin regime are directly complemented by STS. This joint analysis enables a quantitative assessment of how interfacial oxygen reshapes the buried electronic structure and how these modifications manifest at the surface.

**FIGURE 7:**
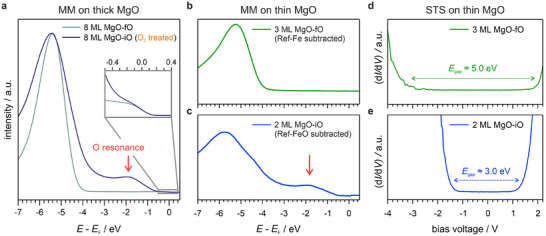
Photoemission and STS data highlighting the electronic impact of the oxygen interlayer. a) EDCs of the 8 ML MgO film grown on Ref‐Fe before (MgO‐fO) and after O_2_ treatment (MgO‐iO), displayed as greyish‐green and dark blue curves, respectively. The inset shows a close‐up of the region around the Fermi energy, where residual intensity from the underlying Fe results in a visible Fermi edge. Upon oxygen treatment, a prominent resonance emerges at −1.8 eV, which lies within the typical MgO bandgap. b),c) EDCs comparing b) a 3 ML MgO‐fO (green) and c) a 2 ML MgO‐iO interface (blue). In b) and c), the background signals of a Ref‐Fe and Ref‐FeO surface were subtracted, respectively. The background‐corrected spectra resemble the ones of the thick 8 ML MgO interfaces, including the resonance at −1.8 eV for the MgO‐iO film. The data presented in a)‐c) were recorded using p‐polarized light at a photon energy of 21.2 eV. d) STS spectrum of the 3 ML MgO‐fO film (without an oxygen interlayer), showing a wide bandgap of 5.0 eV. e) STS spectrum of the 2 ML MgO‐iO film (with a full interlayer of oxygen). The presence of the interlayer results in a significantly reduced bandgap of 3.0 eV.

Figure 7a shows the EDCs for an 8 ML thick film of an MgO interface without an oxygen interlayer (MgO‐fO, grey curve) and with full oxygen intercalation (MgO‐iO, dark blue curve). For the MgO grown on Ref‐Fe, a broad plateau extending up to −4.0 eV below *E*_F_ is observed, consistent with the expected large bandgap of bulk MgO. A faint Fermi edge is visible in the inset due to residual contributions from metallic Fe at the buried interface. After oxygen treatment, a prominent resonance emerges at approximately −1.8 eV, and the main MgO‐related peak located at −5.0 to −6.0 eV broadens. In addition, signatures in the *k*‐resolved Fermi maps reveal a distinct modification of the electronic structure, closely resembling spectral features of the Ref‐FeO surface (see Figure S17 in Section S9).

A similar behavior is observed for thinner (2–3 ML) MgO films, after subtracting the respective reference spectra of Ref‐Fe and Ref‐FeO to account for substrate contributions. The resulting background‐corrected spectra are shown in Figure 7b for MgO‐fO (green curve) and in Figure 7c for MgO‐iO (blue curve), while the non‐corrected spectra can be found in Figure S17 in Section S9. Notably, the background‐corrected spectra reveal the same mid‐gap resonance at −1.8 eV for MgO‐iO, consistent with the thick films.

While states in this energy range have previously been attributed to Mg or O vacancies [53, 54, 55, 56, 57], several observations indicate that a purely defect‐driven interpretation is insufficient. Our AES data confirm the chemical stability of the MgO films, with no evidence of magnesium loss. Furthermore, while annealing below 870 K can lead to the removal of excess O (as discussed for Figure 5a), this process does not degrade the electronic integrity of the layer.

More compellingly, the −1.8 eV state exhibits a well‐defined momentum‐space signature in the momentum microscopy data. As shown in Figure S18 (Section S9), the corresponding *k*‐space features closely resemble the ones that emerge upon oxygen passivation in Ref‐FeO. Such momentum‐space coherence is inconsistent with randomly distributed point defects. While recent work has shown that O vacancy complexes can form structured transport channels in MgO, those states manifest near the Fermi level and drive metallic transport characteristics [58]. In contrast, our observed state appears deeper in the gap and is associated with oxygen presence rather than deficiency.

Complementary insight is obtained from STS measurements. For MgO‐fO (Figure 7d), STS reveals a wide conductance plateau, indicative of a large electronic gap. A pronounced increase in conductance is observed only at approximately −3.0 V for occupied states and near +2.0 V for unoccupied states. Notably, the onset of tunneling at −3.0 V coincides with the emergence of the strong peak in photoemission, underscoring the consistency between both spectroscopies. Together, these observations yield an overall gap size of 5.0 eV. This value agrees well with previous reports on thin films [39, 52], although it remains smaller than the bulk bandgap of 7.6 eV for stoichiometric bulk MgO [39].

The introduction of an O interlayer in MgO‐iO, however, drastically alters the tunneling response (Figure 7e). The conductance onsets shift toward −1.5 eV and slightly below +1.5 eV, resulting in a substantially reduced gap of approximately 3.0 eV. Notably, the onset of tunneling at negative bias coincides energetically with the mid‐gap resonance observed in photoemission. This correspondence suggests that the tunneling response is governed by the interfacial electronic state. While the microscopic mechanism remains open, it may involve evanescent contributions of this interface‐related state, provided its damping across the MgO film is sufficiently weak. While contributions from defect‐mediated tunneling channels cannot be fully excluded, these findings highlight interfacial O engineering as a versatile route to tailor the electronic structure of MgO‐based heterostructures.

## 3 Conclusion

In this study, we demonstrate that interfacial oxygen enables precise control of structural and electronic properties at the MgO/Fe(100) interface while maintaining a high degree of crystalline order. Using MM, LEED, AES, and STS, we establish a reproducible preparation method for oxygen‐free, partially oxidized, and fully intercalated terminations.

Electronic modifications induced by varying oxygen‐concentration regimes persist across increasing MgO thicknesses: although the Fe‐derived intensity around *E*_F_ is strongly suppressed and momentum‐integrated EDCs appear nearly featureless, momentum maps retain clear *k*‐space fingerprints up to 8 ML. This underscores a key advantage of momentum microscopy — its ability to capture subtle reciprocal‐space features even when the energy‐resolved intensity is attenuated by insulating overlayers.

By linking momentum‐resolved photoemission with tunneling spectroscopy, we identify a reproducible electronic resonance inside the MgO gap that emerges upon introducing an interfacial O interlayer. Its distinct appearance in momentum space and its energetic alignment with the onset of tunneling conductance indicate that the tunneling response is sensitive to the buried interfacial electronic structure, while defect‐mediated contributions cannot be fully excluded.

Spin‐resolved momentum maps further reveal that oxygen‐free interfaces suppress minority spin states at *E*_F_, providing spectroscopic evidence of spin filtering at MgO/Fe junctions. In contrast, oxygen intercalation restores spin symmetry, consistent with the commonly reported detrimental effect of interfacial oxygen on the spin polarization at the Fermi level. Beyond this conventional picture, prior work has reported correlation‐driven dynamic spin filtering for oxygen‐passivated Fe(100); VCMA studies in MgO/Fe(100) underscore the functional relevance of interfacial oxygen in device‐like stacks, suggesting that the O interlayer can act as an active tuning parameter for interfacial spin functionality.

These findings demonstrate that spin‐resolved MM is a powerful approach to access spin‐dependent physics at buried oxide/metal interfaces. At the material level, our results establish MgO/Fe(100) as a model platform for atomic‐scale interface engineering in oxide/metal heterostructures. Reproducible control of interface oxidation provides a direct route to optimize spin filtering, TMR, and VCMA in MgO‐based spintronic device stacks.

## 4 Methods

### 4.1 Sample Preparation

For preparing the Fe substrate, a commercial MgO(100) crystal (MaTecK GmbH) was cleaned in vacuum by two cycles of ion sputtering at 2 keV Ar^+^ and subsequent annealing at 870 K for 45 min [41, 59]. A 300 nm thick Fe(100) film was then epitaxially grown in situ by e‐beam deposition on the clean MgO. The resulting surface was further cleaned in the experimental chamber through cycles of ion sputtering (typically 0.5 keV Ar^+^) and annealing (up to 870 K). To prepare Fe(100)‐*p*(1 × 1)O, the Fe surface was passivated by exposing it to O_2_ (30 L) while maintaining a sample temperature of 820 K. After closing the O_2_ leak valve, the sample was annealed at 870 K for 5 min to remove excess oxygen and achieve the desired reconstruction [41].

The MgO films were grown on these substrates using reactive molecular beam epitaxy (MBE). Magnesium pellets with a purity of 99.99% (MaTecK GmbH) were evaporated from a molybdenum crucible using an EFM3 single e‐beam evaporator (Focus GmbH), which was positioned at an angle of approximately 10°–15° with respect to the [010] sample direction. High‐purity oxygen gas (purity grade: > 99.999%) was introduced into the chamber via a leak valve. The base pressure in the chamber prior to growth was approximately 5 × 10^−11^ mbar, and during growth, it increased due to controlled O_2_ flow. The growth process was monitored in situ using reflection high‐energy electron diffraction in medium energy mode (MEED), allowing precise control over film thickness and surface quality. During deposition, samples were kept at T = 440 K following the protocol established by Tekiel et al. [31], who reported improved crystalline quality under these conditions.

In agreement with Tekiel et al., we observed that maintaining this temperature subtly enhanced surface quality compared to room‐temperature growth. However, our results also indicated that both approaches — direct deposition at elevated temperatures or room‐temperature deposition with subsequent annealing — resulted in comparable film qualities. Post‐deposition annealing was carried out under UHV without O_2_ backfilling; during heating, the pressure could transiently increase due to outgassing (up to ∼ 1  ×  10^−9^ mbar). An O_2_ back pressure was applied only for steps explicitly denoted as O_2_ treatment.

### 4.2 Momentum Microscopy (MM, Spin‐MM) and X‐Ray Photoelectron Spectroscopy (XPS)

MM, spin‐MM, and XPS experiments were carried out at the NanoESCA beamline [60] of the Elettra synchrotron (Trieste, Italy). The NanoESCA microscope is equipped with a W(001)‐based spin filter [61], enabling acquisition of constant‐energy spin‐resolved momentum maps over the entire surface Brillouin zone of the system. The photon beam impinged at an angle of 66° with respect to the surface normal along the *k_x_* = 0 direction. Prior to each spin‐resolved measurement, the samples were magnetized along the Fe[001] direction using an oriented permanent magnet placed close to the surface in the analysis chamber. Spin‐resolved data were analyzed following the procedure described in Ref. [62]. Spin‐MM measurements were performed with *p*‐polarized light at a photon energy of 64 eV, while additional MM data with both *p‐* and *s*‐polarization were collected to study light polarization‐dependent effects. The XPS data were acquired with the momentum microscope at a photon energy of 200 and 650 eV. Complementary MM experiments were performed in Dortmund using a Kreios MM by Specs GmbH with a monochromatized UV light source with a photon energy of 21.2 eV (*p*‐polarized light). The MM data recorded in Trieste were collected at 77 K, while MM measurements in Dortmund were performed at room temperature (300 K). To assess potential effects of high‐intensity synchrotron radiation on the MgO sample, we performed repeated scans at a fixed sample position. Within minutes, we observed a shift in the spectrum by several hundred meV, indicating radiation‐induced effects. To prevent radiation‐induced defect formation from biasing the MM measurements, the MgO sample was continuously rastered during data acquisition and moved to a fresh spot for each new measurement. Notably, such radiation‐induced work function shifts were not observed in our laboratory experiments in Dortmund, where rastering of the sample was not necessary. Both momentum microscopes achieve an energy resolution of approximately 100 meV, as determined from analyzing the Fermi‐edge line shapes. EDCs were obtained by integrating the momentum maps over the entire visible momentum range of [*k*_x_, *k*_y_] ∈[−2.0,2.0] Å^−1^.

The XPS Data Were Analyzed Using the XPS Tools (XPST) Package for IGOR Pro [63]

Both MM setups in Dortmund and Trieste are equipped with almost identical preparation chambers. Therefore, the following method sections (AES, LEED, and MEED) apply to both setups.

### 4.3 Auger Electron Spectroscopy (AES)

Auger electron spectroscopy data were recorded using a DESA150 system from Staib Instruments, equipped with a cylindrical mirror analyzer (CMA). The setup was operated in lock‐in mode to reduce noise and provide differential intensity spectra.

### 4.4 Low‐Energy Electron Diffraction (LEED) and Medium Energy Electron Diffraction (MEED)

LEED data were recorded using a LEED ErLEED 150 system from Specs. MEED measurements were performed using the electron gun of the oppositely mounted Staib Instruments Auger setup, where the electron beam was directed at the sample at an angle indicated in Figure S1. The diffracted beams were then monitored on the LEED screen.

### 4.5 Scanning Tunneling Microscopy (STM) and Scanning Tunneling Spectroscopy (STS)

STM and STS measurements were performed using an Omicron LT‐STM system at CNR—Istituto Officina dei Materiali (IOM) in Trieste, Italy, under UHV conditions (base pressure < 7 × 10^−11^ mbar). The data were recorded at a temperature of 77 K to ensure stability and precision in imaging and spectroscopy. Topographic STM images were recorded in constant‐current mode. STS spectra were collected with a lock‐in amplifier (modulation frequency 975 Hz, amplitude 16 mV). The MgO/Fe interfaces for the STM studies were prepared and characterized in Dortmund (Germany). Samples were then transferred to Trieste (Italy) using a Ferrovac UHV suitcase (base pressure below 5 × 10^−11^ mbar during transfer). The suitcase was connected to the STM chambers via fast‐entry load locks, which were baked out and allowed to cool down before sample transfer. During transfer into the load lock, samples were briefly exposed to pressures of up to 5 × 10^−8^ mbar.

### 4.6 X‐Ray Absorption Spectroscopy (XAS)

XAS measurements were carried out in total electron yield mode at the X‐Treme beamline [64] of the Swiss Light Source (SLS), Paul Scherrer Institute (PSI), Villigen, Switzerland. While the sample with the sharp MgO‐fO interface (without O interlayer) was transferred via a vacuum suitcase (see prior description in the STM and STS methods), the MgO‐iO interfaces (with O interlayer) were grown on‐site using the local preparation chamber. To ensure reproducibility for the growth, the Mg evaporator from Dortmund was moved and mounted to this preparation chamber. A combined LEED/Auger ErLEED by Specs GmbH was used to monitor the chemical composition alongside a quartz crystal microbalance (QCM) to initially calibrate the Mg deposition rate.

### 4.7 Density Functional Theory (DFT)

We performed density functional theory (DFT) calculations using VASP 6.4.1. [65, 66, 67, 68, 69, 70], where we employed a repeated slab approach with six layers of Fe with (MgO‐iO) and without O‐passivation of the topmost layer (MgO‐fO), two ML of MgO, and a 25 Å vacuum layer between periodic images of the slab. For the geometry optimization, we allowed all atoms to relax, except for the bottom two Fe layers, while using the PBE‐GGA functional [71] with van‐der‐Waals corrections according to the Grimme‐D3 scheme with a Becke–Jones damping [72, 73]. The reciprocal lattice was sampled with a Γ‐point centered 10 × 10 × 1 grid for the geometry optimization and 20 × 20 × 1 grid for a final single‐shot calculation for the electronic structure analysis.

## Conflicts of Interest

The authors declare no conflicts of interest.

## Supporting information




**Supporting file**: advs74496‐sup‐0001‐SuppMat.pdf.

## Data Availability

The data that support the findings of this study are openly available in Zenodo at 10.5281/zenodo.18331593, reference number [18331593].
