# Supplementary material for: Spin‐Selective Interface Engineering in Oxide–Ferromagnetic Junctions via Atomic‐Scale Oxygen Control
Source: Adv Sci (Weinh). 2026 Feb 20;13(25):e23165. doi: 10.1002/advs.202523165 (PMC13137816; doi:10.1002/advs.202523165)
Supplement: Supplementary file 1 — Supporting file: advs74496‐sup‐0001‐SuppMat.pdf. [file ADVS-13-e23165-s001.pdf]

Supporting Information: Spin-Selective Interface Engineering in Oxide –Ferromagnetic Junctions via Atomic-Scale Oxygen Control

David Maximilian Janas[1, *], Mira Sophie Arndt[1], Jonah Elias Nitschke[1], Lasse Sternemann[1], Valentin Mischke[1], Vitaliy Feyer[2], Iulia Cojocariu[2], Daniel Baranowski[2], Alessandro Sala[3], Andreas Windischbacher[4], Peter Puschnig[4], Jan Dreiser[5], Stefano Ponzoni[1], Giovanni Zamborlini[1,4, #] and Mirko Cinchetti[1].

[1]*TU Dortmund University, Department of Physics, 44227 Dortmund, Germany.*

[2]*Peter Grünberg Institute (PGI-6), Forschungszentrum Jülich GmbH, 52428 Jülich, Germany.*

[3]*CNR – Istituto Officina dei Materiali (IOM), S.S. 14 km 163.5, Area Science Park, Basovizza, Trieste, 34149 Italy.*

[4]*Karl-Franzens-Universität Graz, Institut für Physik, NAWI Graz, 8010 Graz, Austria.*

[5]*Swiss Light Source, Paul Scherrer Institute, 5232 Villigen PSI, Switzerland.*

Corresponding authors: * david.janas@tu-dortmund.de; # giovanni.zamborlini@uni-graz.at

Supporting information

S1: MEED

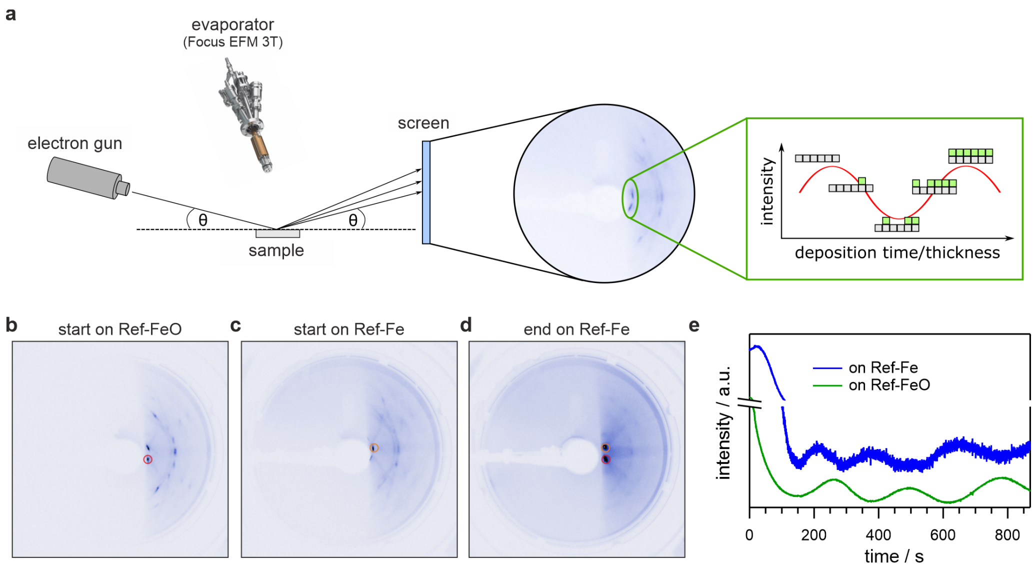

Figure S1: *MEED measurements of MgO growth on Fe(100).* ***a)*** *Schematic illustration of a MEED experiment. Tracking the intensity oscillations of the diffraction spots allows precise calibration of the ML coverage for layer-by-layer growth. The evaporator shown is a Focus EFM 3T model.* ***b)-d)*** *MEED images of* ***b)*** *Fe(100)-p(1 × 1)O (Ref-FeO),* ***c)*** *pristine Fe(100) (Ref-Fe), and* ***d)*** *2 ML of MgO grown on Ref-Fe. Notably, the MEED images at the end of the growth were identical for MgO grown on Ref-Fe and Ref-FeO (not shown here). Tracking the intensity of the highlighted spots yields the oscillating curves presented in* ***e)****.*

In our study, we focus on the growth on two different substrates: 1) clean Fe(100) (Ref-Fe), and 2) oxygen-passivated Fe(100)-*p*(1 × 1)O (Ref-FeO) under varying conditions. To monitor both the thickness and the crystalline quality during growth, we employed the MEED method. This technique allows precise calibration of the deposition rate and enables real-time adjustment of the growth duration to achieve nearly perfect monolayer coverage (see Fig. S1). To ensure high crystalline quality during the MEED

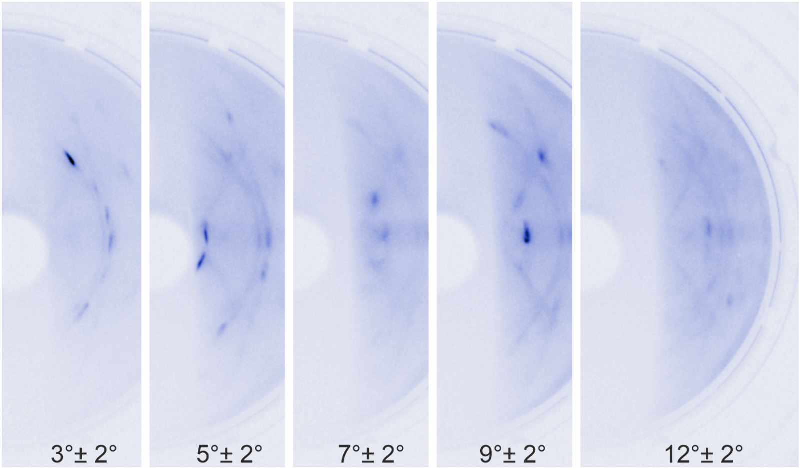

Figure S2: *Representative MEED images of an Fe(100) surface captured at various angles with a kinetic energy of 5 keV. Optimal MEED oscillations were consistently observed at an incident angle θ of approximately 5°.*

recordings, the oxygen back pressure was maintained in the range of 2×10^{-8} mbar to 5×10^{-8} mbar (for reference: low pressure was typically 5×10^{-9} mbar), while the sample was kept at T = 440 K. In addition, we found that an incident angle of θ = 5° worked best for tracking the MEED oscillations during film growth. As shown in Figure S1, at this angle the diffraction spots corresponding to the Ref-Fe, Ref-FeO, and the MgO/Fe interfaces overlap, providing a strong and easily detectable signal. For diffraction spots at other angles (see Fig. S2), oscillations were more difficult to track reliably, likely due to weaker intensity and poorer overlap of the relevant diffraction features. Therefore, we consistently used the 5° geometry for all MEED-based growth monitoring.

A notable difference in the monitored intensity curves was observed for the two surfaces: while on Ref-FeO (green curve) the intensity drops monotonically until the first peak, on clean Ref-Fe (blue curve) we consistently observed an initial rise (typically at times between 30-100 s).

One possible explanation for this early rise on pristine Ref-Fe is the formation of the passivating oxygen layer that develops at the very beginning of the growth process. The precise timing of this feature depends on the oxygen background pressure and on the availability of surface oxygen, for example, from residual contamination. In contrast, this initial rise is absent for growth on Fe-O, where the surface is already saturated with oxygen before Mg deposition starts.

Further insight into this phenomenon is provided by Oh *et al.*[1], who showed that clean Fe surfaces do not spontaneously oxidize under comparable oxygen pressures and temperatures unless heated to much higher temperatures (770 K). At room temperature, oxygen uptake only occurs when Mg is present — demonstrating that Mg atoms act catalytically by promoting oxygen dissociation and surface passivation. This process is initiated when the Mg evaporator shutter is opened, releasing Mg atoms that readily donate electrons to adsorbed O_2 molecules, filling antibonding orbitals and inducing dissociation into atomic oxygen. The released oxygen atoms then react with surface Fe, forming an initial Fe-O layer while MgO simultaneously begins to nucleate. Our observations align with these findings.

This mechanism explains the absence of the initial intensity rise on Ref-FeO: since the Ref-FeO surface is already oxygen-saturated, no catalytic activation is required, and growth proceeds directly into MgO formation. On clean Ref-Fe, however, the rise reflects this transient passivation process, which competes with the early stages of MgO film formation. This initial oxidation can be partially reversed by post-growth annealing, which promotes oxygen transfer from the passivated Fe-O into the MgO layer, but often some residual Fe-O remains.

S2: Linear Dichroism Effects in ARPES and XAS

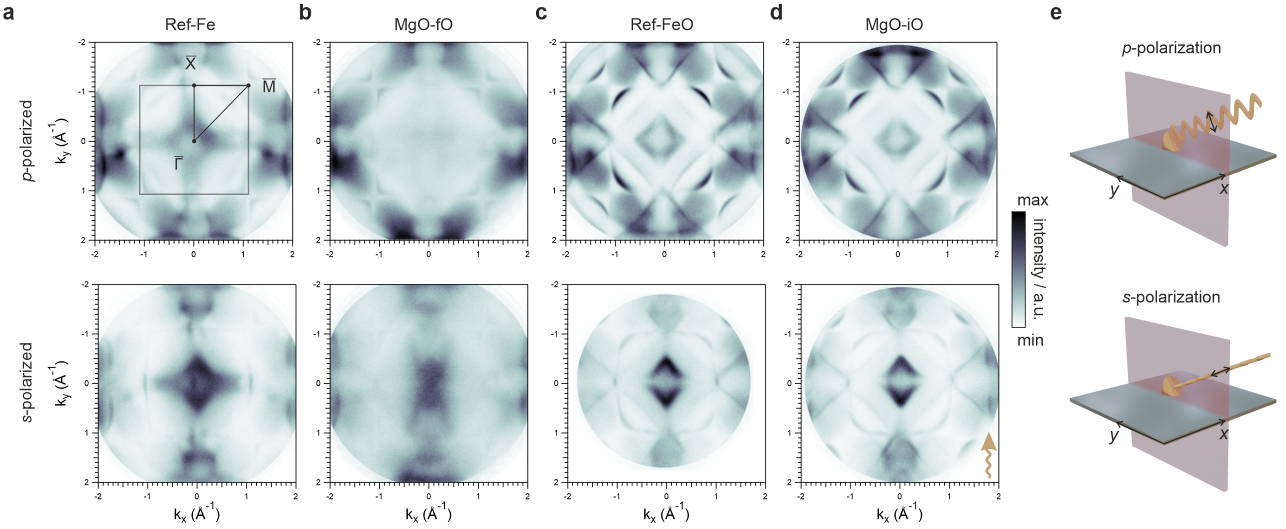

Figure S3: Momentum maps at the Fermi energy for ***a)*** *Ref-Fe,* ***b)*** *MgO-fO (without O interlayer),* ***c)*** *Ref-FeO, and* ***d)*** *MgO-iO (with O interlayer). The top row shows images taken using p-polarized and the bottom images taken with s-polarized light. The experimental geometry is presented in* ***e)****. The grey square in a) indicates the 1st surface Brillouin zone (SBZ) of Fe(100). All presented maps were recorded at a photon energy of 64 eV.*

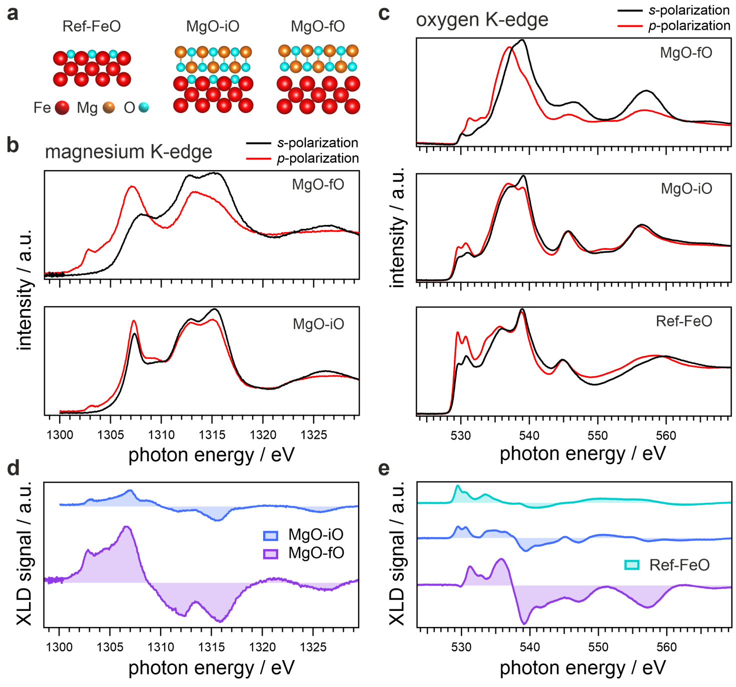

Figure S4*: XAS data of different Fe and MgO/Fe surfaces.* ***a)*** *Structural models of Ref-FeO and MgO-iO and MgO-fO. XAS spectra of the Mg K-edge and the O K-edge are presented in* ***b)*** *and* ***c)****, respectively. In* ***d)*** *the linear dichroism observed for the measurements at the Mg K-edge is reported, and in* ***e)*** *the linear dichroism at the oxygen K-edge. The data were recorded under 70° grazing incidence while the samples were kept at room temperature (300 K).*

S3: Identification of Features in the Band Structure

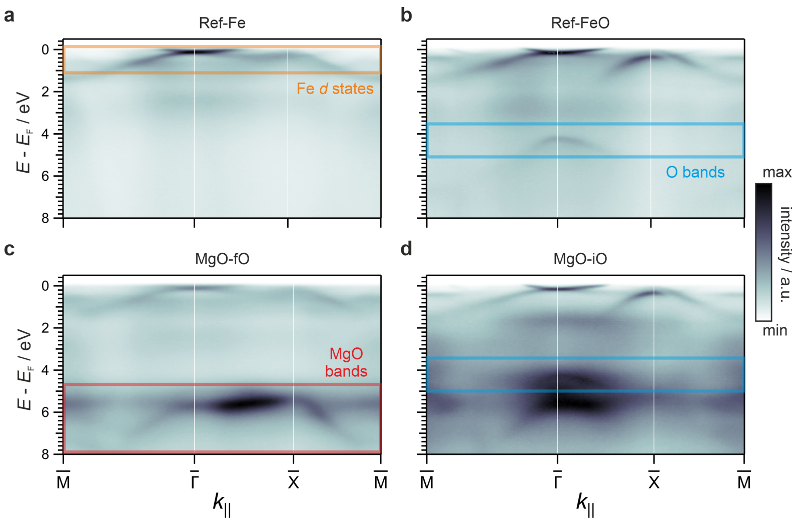

Figure S5: *Experimental band structure comparison of **a)** Ref-Fe, **b)** passivated Ref-FeO, **c)** MgO-fO (without O interlayer), and **d)** MgO-iO (with O interlayer). Prominent bands are highlighted according to their electronic origin. Note that these cuts were recorded using s-polarized light (64 eV photon energy). The band structure cuts were extracted along the high-symmetry directions of Fe(100), indicated in Fig. S3.*

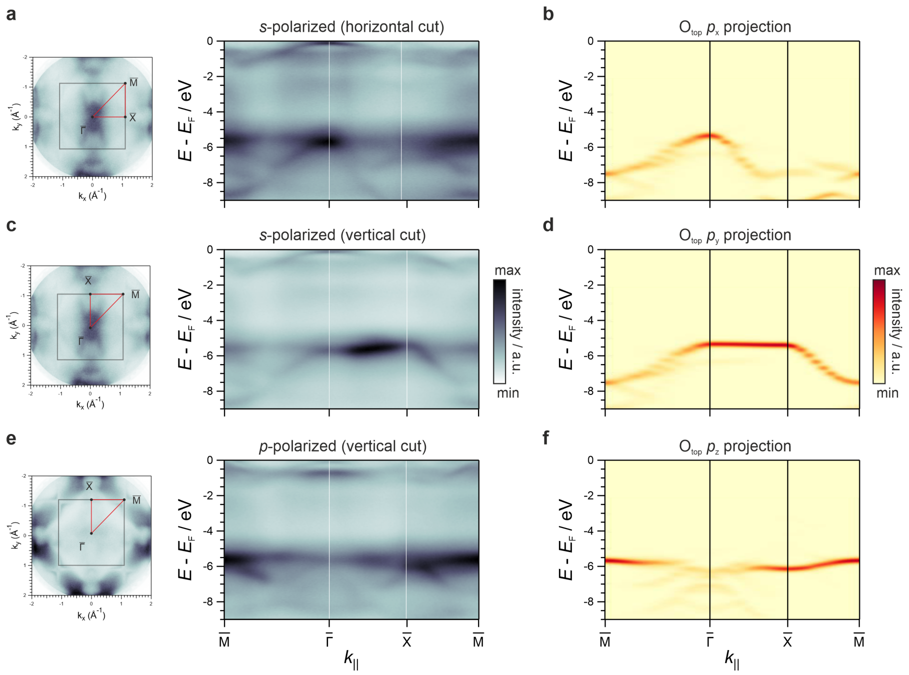

Figure S6: *Polarization- and direction-dependent band cuts used for qualitative identification of MgO-derived bands for MgO-fO. Panels **a),c),e)** show experimental Fermi maps (for the indicated light polarizations) with the symmetry-equivalent orthogonal cut directions marked; the corresponding extracted band dispersions are displayed in the adjacent panels (recorded using hν = 64 eV). Owing to dipole selection rules, the combination of light polarization and cut direction enhances sensitivity to different orbital characters. Panels **b),d),f)** show the corresponding DFT band structure projected onto the O 2p orbitals of the topmost (surface) MgO layer in a model consisting of 2 ML MgO on a 6-layer Fe(100) slab. For visual alignment to the experimental energy scale, the calculated MgO-derived valence-bands are rigidly shifted by -2.0 eV to account for the underestimated MgO gap.*

S4: Fit Results of the Fe 3*p* and Mg 2*p* Core Levels and XPS Data of the O 1*s* and O 2*s* Core Levels

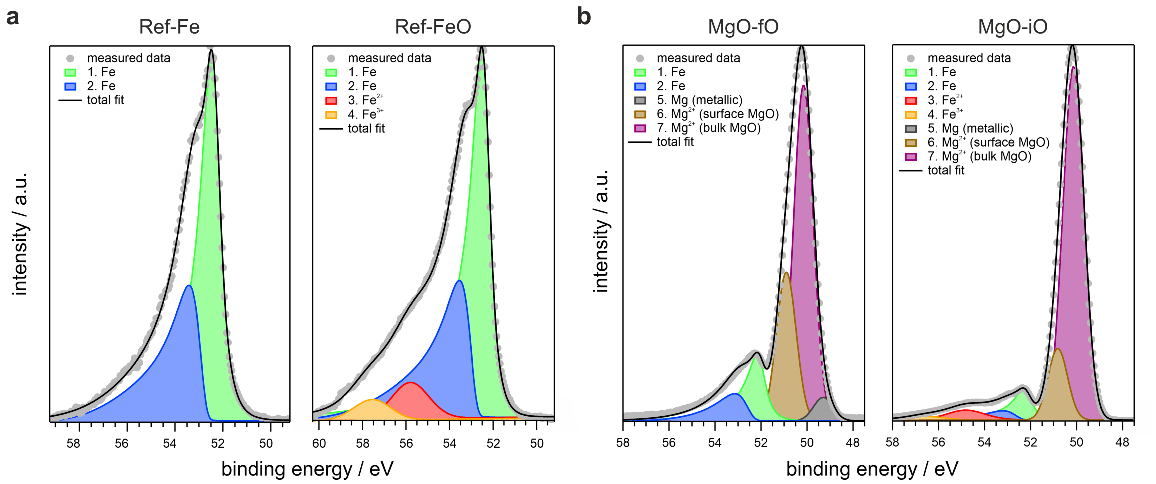

Figure S7: *XPS analysis of the Fe 3p and Mg 2p levels during different stages of MgO growth.* ***a)*** *XPS spectra of the Fe 3p level for Ref-Fe (left) and Ref-FeO (right). The Ref-FeO system exhibits additional shoulders compared to Ref-Fe, attributed to* Fe^{2+} *and* Fe^{3+} *valence states resulting from strong chemical bonding between O and Fe surface atoms.* ***b)*** *XPS spectra for two exemplary 2 ML MgO films grown on Fe(100): one at low* O_2 *pressure (left) and the other one on Fe-O at high* O_2 *back pressure (right). The spectrum on the left corresponds to an MgO-fO interface, while the spectrum on the right represents an MgO-iO interface featuring a full intercalated layer of oxygen. The energy range includes both the Fe 3p and the Mg 2p levels. For clarity, a Shirley background has been subtracted from all spectra. Data were recorded using a photon energy of 200 eV.*

Figure S7a compares XPS spectra of the Fe 3*p* level for clean Ref-Fe (left) and oxygen-passivated Ref-FeO (right). On Ref-Fe, two asymmetric features characteristic of metallic Fe are observed. This complex shape reflects an unresolved sextuplet structure of the Fe 3*p* core level, arising from spin-orbit coupling effects.[2] In contrast, additional peaks emerge on the passivated surface, corresponding to Fe^{2+} and Fe^{3+} valence states. These features result from strong chemical bonding between oxygen atoms and surface Fe atoms,[3] providing evidence for interfacial oxidation.

To quantitatively describe the metallic Fe 3*p* peak, it was fitted using two asymmetric pseudo-Voigt functions.[4] The additional shoulders attributed to Fe^{2+} and Fe^{3+} states were modeled separately using symmetric pseudo-Voigt functions.

Fig. S7b shows XPS spectra for two representative MgO films: one grown at low O_2 pressure on pristine Ref-Fe (left), and another grown at high O_2 pressure on oxygen-passivated surfaces, Ref-FeO (right). For these spectra, the metallic Fe contributions were constrained to match those of the clean substrates, with only minor adjustments permitted during the fitting. This approach preserves the line shape of the Fe 3*p* level while allowing isolation of the new spectral features associated with Mg 2*p*. In contrast, the energy positions of the Fe^{2+} and Fe^{3+} peaks were left unconstrained to account for possible shifts induced by the interface formation. For the Mg 2*p* level, three distinct Gaussian components were introduced: contributions from bulk-like MgO,[5] surface-related MgO[6] — previously reported for MgO grown on Mo(100) — and a small metallic Mg component[7] that vanishes entirely in samples with a fully intercalated oxygen layer. A summary of the fit results is given in Table S1. Notably, neither the main metallic Fe nor the bulk-like Mg peak shifts significantly across these samples. However, films with higher oxygen concentration exhibit additional features near the binding energies associated with Fe^{2+} and Fe^{3+} states,[8,9] consistent with the presence of intercalated oxygen at the interface.

***Table S1**: Fit results of the XPS fits for the Mg 2p and Fe 3p core levels presented in Fig. S7.*

		Ref-Fe	Ref-FeO	MgO-fO	MgO-iO
peak #1 Fe	rel. area / a.u.	1317.51	221.37	140.57	136.17
	position / eV	52.58	52.7	52.31	52.43
peak #2 Fe	rel. area / a.u.	671.94	110.69	68.55	48.99
	position / eV	53.78	53.98	53.36	53.42
peak #3 Fe^{2+}	rel. area / a.u.		25.06		63.05
	position / eV		55.8		54.82
peak #4 Fe^{3+}	rel. area / a.u.		14.41		23.94
	position / eV		57.66		56.57
peak #5 Mg	rel. area / a.u.			31.76	1.38
	position / eV			49.3	49.1
peak #6 Mg^{2+} (surf)	rel. area / a.u.			202.72	202.94
	position / eV			50.9	50.82
peak #7 Mg^{2+} (bulk)	rel. area / a.u.			457.69	1084.31
	position / eV			50.16	50.13

To further investigate the oxygen environment, the O 1*s* and O 2*s* core levels were examined. The O 1*s* peak in Figure S8 shows no significant shift between the pristine Ref-FeO surface and MgO-pO interfaces, with binding energies consistently centered around 530.1 eV. This indicates that the oxygen atoms are similarly bound in both cases — either directly to Fe in terms of an Fe–O layer or within the MgO lattice. Hence, the oxygen-related XPS peaks alone are not particularly sensitive to subtle changes in the interfacial chemical composition.

A similar conclusion is drawn from the O 2*s* peak data shown in Figure S9, where an MgO-iO film (with O interlayer) is compared to a sharp MgO-fO interface. The spectra are normalized to the Mg 2*p* peak (not shown in Fig. S9), ensuring that any difference in the O 2*s* peak area directly mirrors differences in relative O content. If the interlayer system (MgO-iO) consisted of 2 ML of MgO plus a monolayer of interfacial oxygen, the O 2*s* peak area would be expected to be approximately 1.5 times that of the sharp MgO-fO interface. However, the observed O 2*s* area is nearly twice as large, indicating a higher O concentration than expected. This suggests that, in addition to the interfacial oxygen layer, further O from undefined sources may contribute to the overall signal. The fit results for the O 1*s* and O 2*s* regions are summarized in Table S2 and Table S3, respectively.

All XPS fits were performed using the XPST analysis tool described in [10], which utilizes asymmetric Pseudo-Voigt functions. Given that our primary goal here is to extract quantitative peak parameters (positions, areas, widths), this approach is well-suited for the present analysis.

***Table S2**: Fit results of the XPS fits for the O 1s core level spectra presented in Fig. S8.*

	Ref-Fe	Ref-FeO	2 ML MgO-pO (as dep.)	2 ML MgO-pO (870 K flash)
**position / eV**	530.15	530.04	530.16	530.07
**FWHM / eV**	0.90	1.00	1.46	1.47
**GL-ratio**	0.3	0.5	0.3	0.3
**asymmetry**	0	0.47	0	0

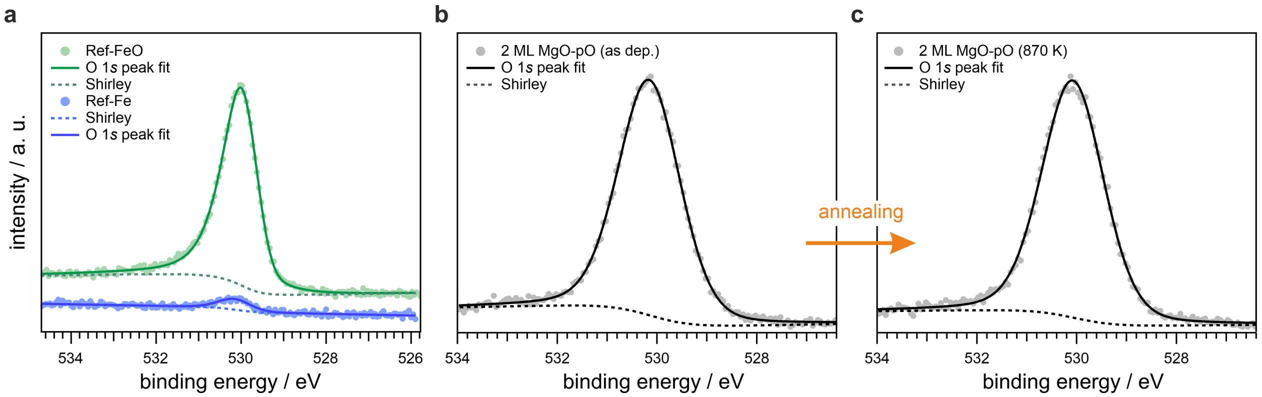

***Figure S8**: Comparison of different fits of the O 1s core level for **a)** Ref-Fe (blue) and Ref-FeO (green), **b)** a 2 ML MgO-pO (grown on Ref-FeO but at low O_2 pressure) before post-deposition annealing, and **c)** after flash annealing to 870 K. Measurements were recorded using a photon energy of 650 eV.*

Table S3: XPS fit results of the O 2s spectra shown in Fig. S9 and the corresponding Mg 2p peaks (not shown). The interfaces correspond to one sample with a sharp MgO-fO interface and one with a fully intercalated oxygen layer (MgO-iO).

		MgO-fO	MgO-iO
O 2*s* peak	norm. area / a.u.	1.00	1.95
	position / eV	22.09	22.00
	FWHM / eV	2.25	2.10
Mg 2*p* peak	position / eV	50.04	50.00
	FWHM / eV	1.22	1.15

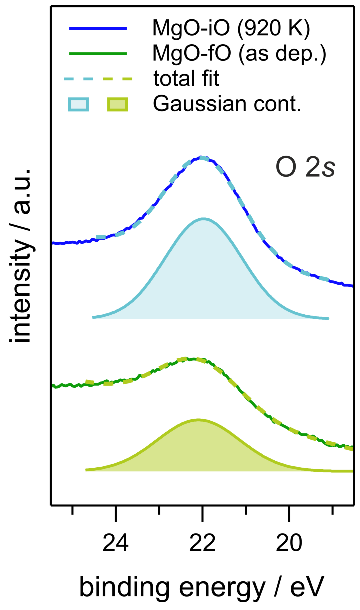

***Figure S9**: Supporting XPS data of the O 2s core level for MgO films with and without an oxygen interlayer (O interlayer verified by MM). The spectra were recorded using 200 eV photon energy. The blue curve corresponds to an MgO-iO film (with an oxygen interlayer) after high-temperature annealing, while the green curve represents an MgO-fO interface (free of interfacial oxygen) before annealing. Although the O 2s peak area is significantly larger for the MgO-iO sample, no notable shift in binding energy or variation in peak width is observed. The peaks were fitted by the sum of a Gaussian and a linear background. The spectra are normalized to the Mg 2p peak, which is not displayed.*

S5: Influence of Oxygen Passivation on the MgO Growth

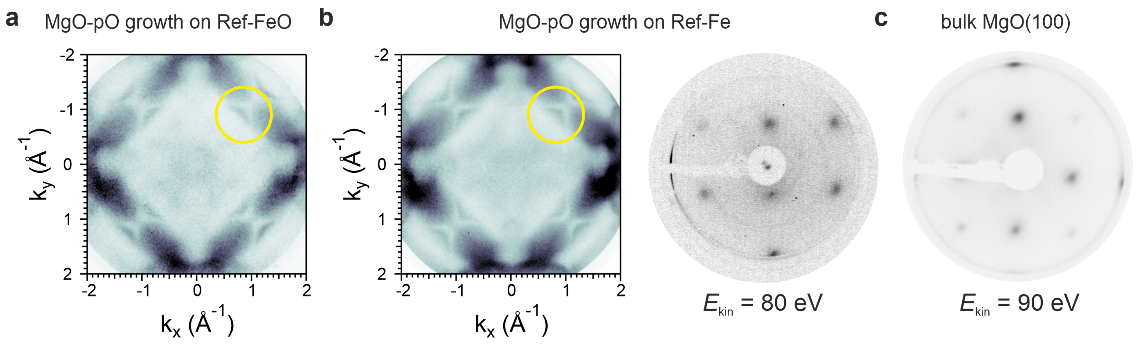

***Figure S10**: **a)** Momentum map at the Fermi energy of a 2 ML MgO-pO thin film grown on Ref-FeO. **b)** Momentum map at the same energy for MgO-pO grown on clean Ref-Fe (left), accompanied by a LEED image (right) of the same sample. A specific feature, previously associated with oxygen, is highlighted in the photoemission maps (hν=64 eV, p-polarized). The films were grown at comparable medium O_2 background pressures. The subtle differences between the two maps suggest a higher presence of interfacial O when growth occurs on Ref-FeO. This is supported by the measured work functions: 2.7 eV for the MgO-pO film grown on Ref-Fe, and 2.9 eV for the one grown on Ref-FeO. Unlike the MgO-fO films prepared on Ref-Fe under low O_2 back pressure (see Fig. 4e), this LEED pattern does not show elongated spots. This indicates that both films presented here (grown under medium O_2 back pressure and annealed at 870 K after growth) are influenced more by the total oxygen availability than by surface-specific growth modes. **c)** LEED image of a bulk MgO(100) crystal.*

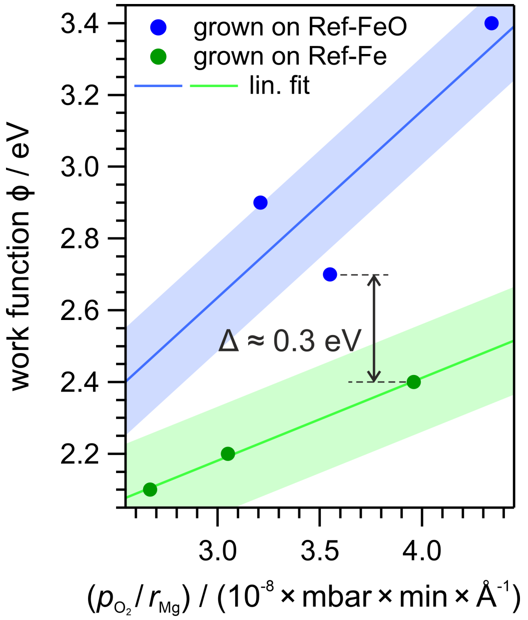

***Figure S11**: Work function of selected MgO/Fe interfaces grown on Ref-FeO (blue markers) and Ref-Fe (green markers) as a function of the ratio of O_2 pressure to Mg deposition rate (p/r). Linear fits highlight differences in slope between the two growth conditions, with an approximate offset of 0.3 eV to higher work functions for samples grown on Ref-FeO under similar conditions. This suggests that pre-passivating the surface with oxygen increases the availability of oxygen atoms during growth. The presented p/r range represents optimal conditions for producing sharp MgO-fO interfaces with minimal defects. Notably, our optimized p/r value is approximately half of that reported by Tekiel et al., who focused on crystalline quality at the MgO surface rather than interface properties.*

S6: Comparison of Structural and Electronic Properties

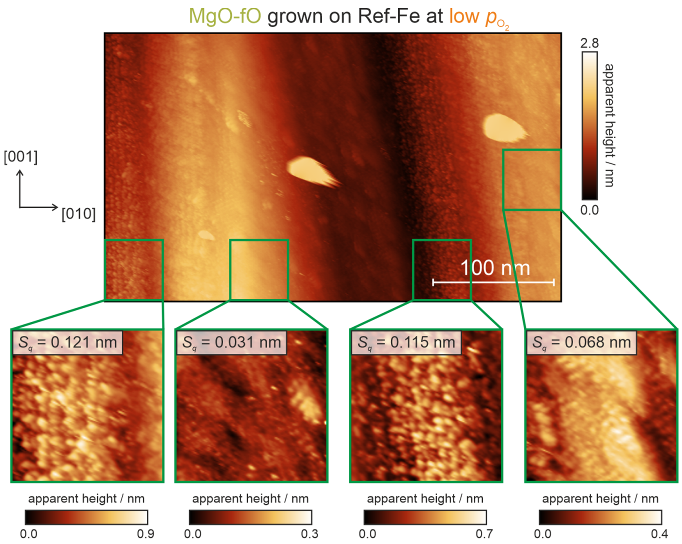

***Figure S12**: STM morphology and local roughness analysis of MgO-fO grown at the lowest oxygen pressure. The upper panel reproduces the STM topograph shown in Fig. 4f. The four lower panels display magnified views of selected regions, highlighting alternating granular and comparatively smooth patches. In the low-oxygen-growth limit, the granular patches are consistent with locally increased disorder and may reflect regions of non-ideal stoichiometry (e.g., locally reduced oxygen content), although STM alone does not provide direct chemical sensitivity. The local areal root-mean-square (RMS) height roughness S_q was evaluated for these 50 × 50 nm² sub-areas (plane-leveled) and is indicated in the insets. The two isolated protrusions with a lateral size of about 30 nm are attributed to particulate contamination.*

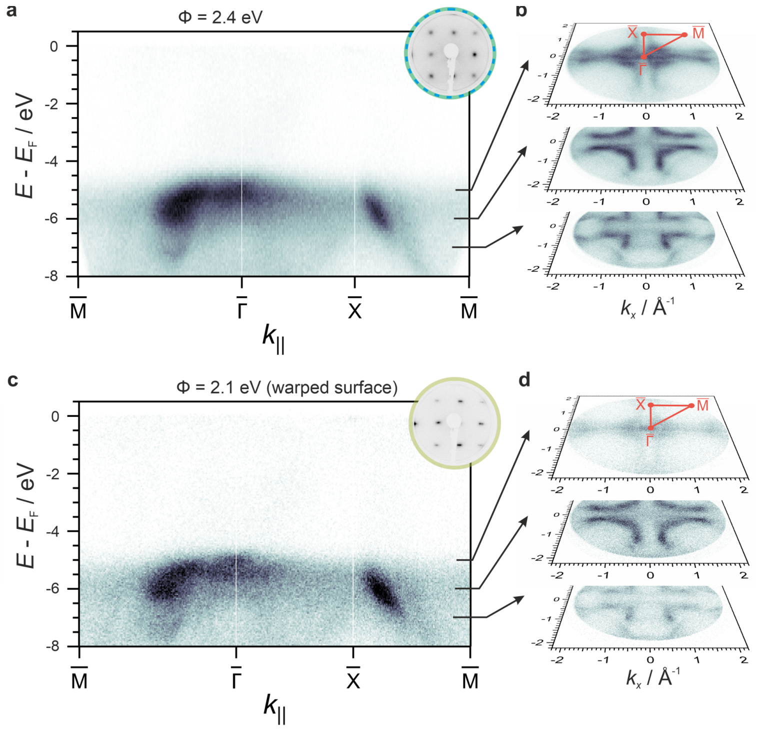

***Figure S13**: MM measurements of MgO-fO films grown under different oxygen pressures, comparing electronic structure and surface morphology. After growth, both MgO-fO films discussed here were post-annealed to 870 K under identical conditions. Panels **a)** and **b)** show a film grown at low O_2 pressure, yet sufficient to form a well-ordered film, where the LEED pattern (inset) exhibits uniform spots and the corresponding band dispersion reveals well-defined MgO-derived features; the constant-energy maps in b) are taken at the indicated energies and highlight these MgO bands. Panels **c)** and **d)** present an MgO film grown at reduced oxygen pressure, for which LEED and STM (not shown) indicate a warped surface; nevertheless, the band dispersion in c) and the associated constant-energy maps in d) demonstrate that the MgO-related electronic structure remains qualitatively similar to the film in a). The data were recorded using p-polarized light at 21.2 eV.*

S7: Auger Electron Spectroscopy and Thickness Evaluation

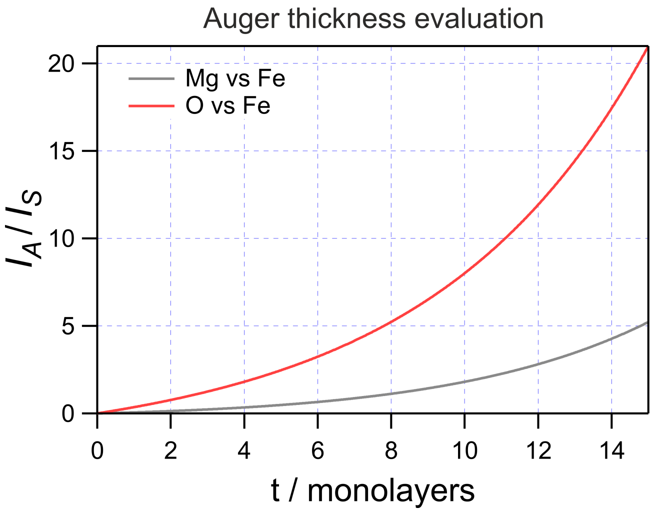

Figure S14: *Thickness calibration curves for MgO barriers, calculated based on the measured peak-to-peak ratios of Mg vs. Fe (grey curve) and O vs. Fe (red curve) Auger intensities. These curves allow precise estimation of the MgO film thickness from experimental Auger data. The calculations are based on parameters provided in Table S4 that are described in detail in the text.*

The curves for thickness calibration presented in Fig. S14 were calculated using the following formula:[11]

$$\frac{I_A}{I_S} = \frac{S_A}{S_S} \cdot \frac{1 - \exp\left(-\frac{t}{\cos(42°) \cdot \lambda_A}\right)}{\exp\left(-\frac{t}{\cos(42°) \cdot \lambda_S}\right)},$$

where t is the thickness of the grown barrier, and I_A and I_S are the measured peak-to-peak ratio of the adsorbate (Mg and O) and the substrate (Fe) peaks, respectively. The parameters S_A and S_S denote the sensitivity factors of the adsorbate and substrate peaks, while λ_A and λ_S are the inelastic mean free paths corresponding to the kinetic energies of the respective Auger peaks. The angle of 42° is specific to the geometry of the Auger electron spectrometer used in the experiments. Based on this approach, effective layer thicknesses were independently determined from the O/Fe and Mg/Fe ratios. Assuming a homogeneous film, the relative oxygen content was then estimated via the ratio $t_{\mathrm{O}}/(t_{\mathrm{O}} + t_{\mathrm{Mg}})$, providing a quantitative measure of stoichiometry. For thick MgO films (8 ML) grown under sufficient O_2 back pressure, this method yields a value of 0.52, in good agreement with the ideal value of 0.5 for stoichiometric MgO.

Table S4: *Parameters used for thickness calibration of MgO films grown on Fe. The table lists the relevant elements (Mg, O, Fe), the investigated Auger transitions with their corresponding kinetic energies, sensitivities (from Ref. [12]), and inelastic mean free paths (λ) interpolated from Shinotsuka et al.[13] A thickness of one MgO monolayer (ML) corresponds to 2.105 Å.*

element	Auger transition	E_{kin} / eV	sensitivity S	IMFP λ / nm
Mg	KLL	1174	0.3658	2.51
O	KLL	503	1.2571	1.37
Fe	$L_{2,3}M_{2,3}M_{4,5}$	654	0.7878	1.62

S8: Influence of Post-Deposition Annealing

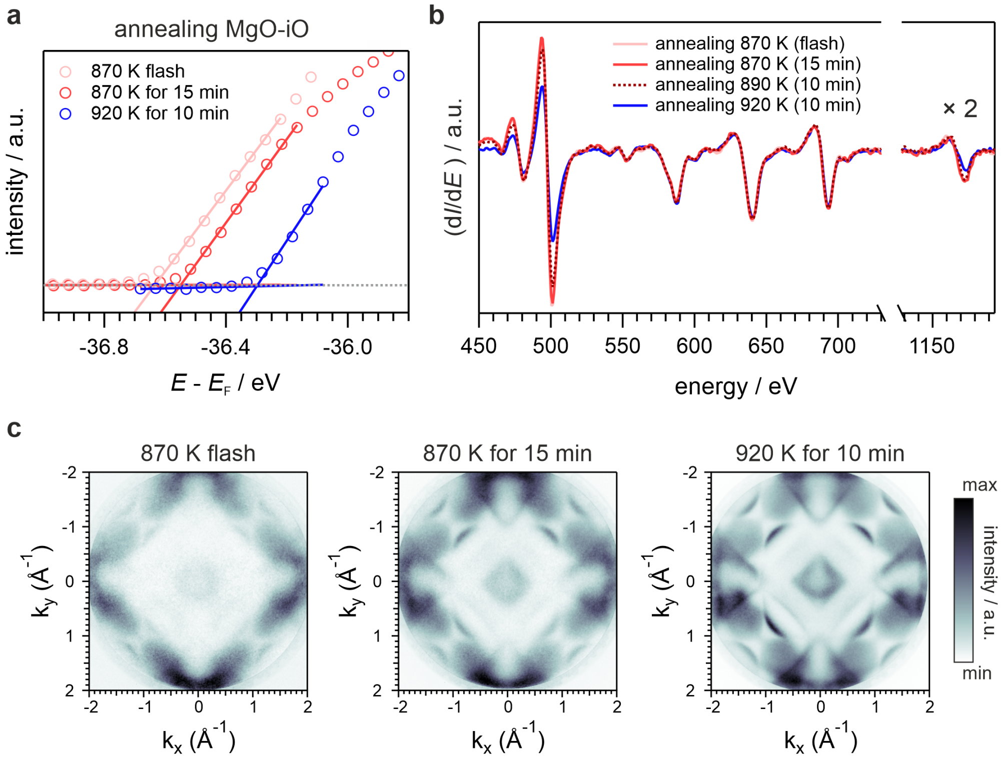

Figure S15**: Influence of annealing on MgO-iO films grown under high O_2 back pressure on Ref-FeO.* ***a) *For prolonged annealing at temperatures higher than 870 K, a shift of the secondary cut-off towards E_F is observed, corresponding to an increase of the system's work function.* ***b)*** *Auger spectra for the various annealing steps indicate a change in the chemical composition, especially a reduction of Mg and O intensities for the higher annealing steps. The spectra are normalized to the Fe-related features.* ***c)*** *Momentum maps recorded after different annealing steps, showing how the fingerprints of the Fe-O interface become more pronounced (hν=64 eV, p-polarized).*

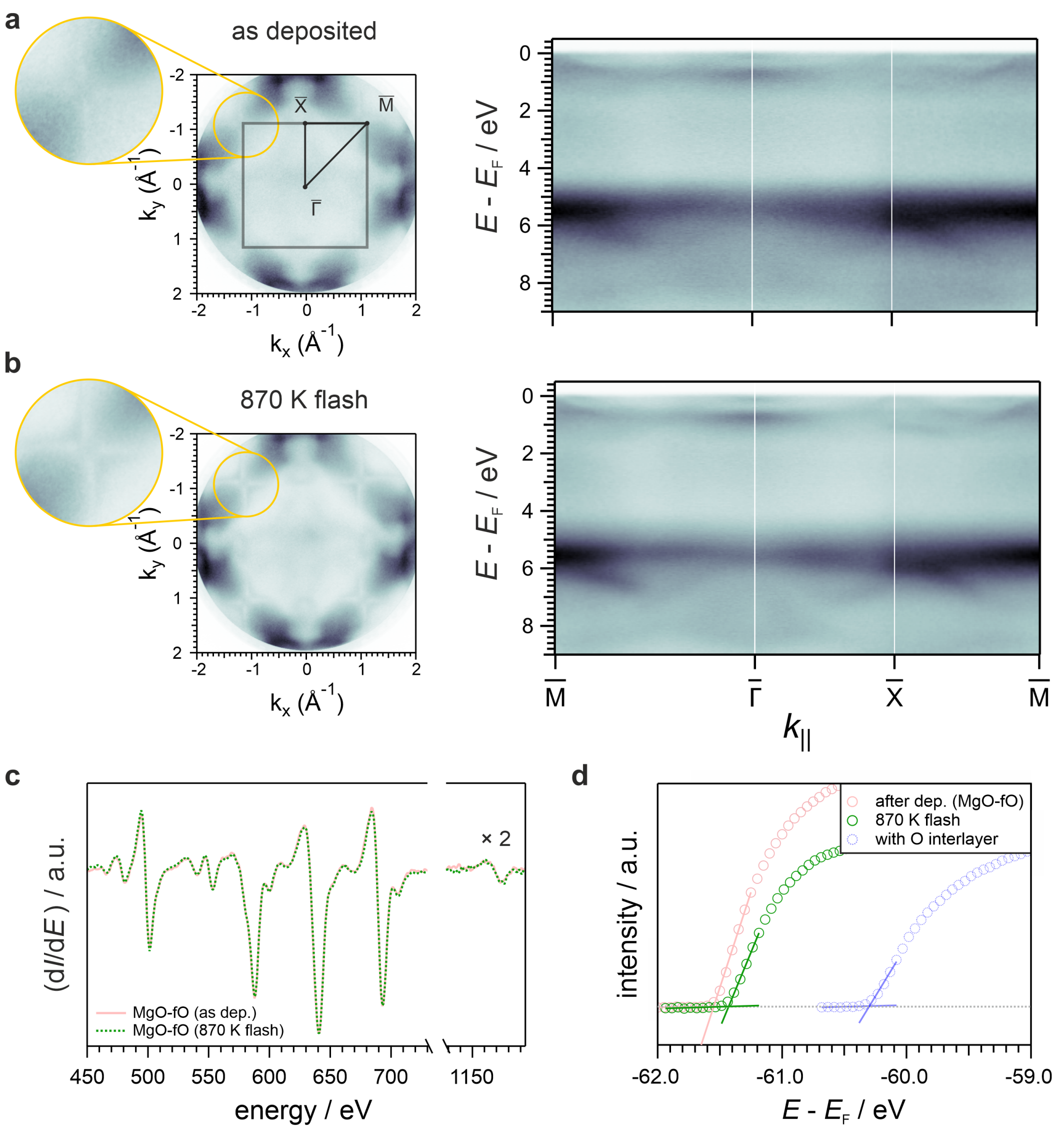

__Figure S16__: Influence of the flash-annealing on low-pressure MgO-fO films grown on Ref-Fe. __a)__ Momentum map of the MgO-fO system right after deposition (left) and the corresponding band structure (right). The band structure is extracted following the high symmetry lines (black). __b)__ Maps and band structure of the same system, but after flash annealing to 870 K. Comparing the data in a) and b) demonstrates a sharpening of bands and k-features upon annealing. The photoemission measurements were recorded using p-polarized light at a photon energy of 64 eV. __c)__ Auger spectra of the MgO-fO film before (light red) and after post-deposition annealing at 870 K (dotted green). __d)__ Illustration of the variation in the secondary cut-off of the MgO-fO interface due to annealing. For a better overview, an exemplary secondary cut-off for an unrelated MgO-iO film with O interlayer is superimposed as well (dotted blue).

S9: Momentum-Resolved Data for Different Thicknesses of MgO

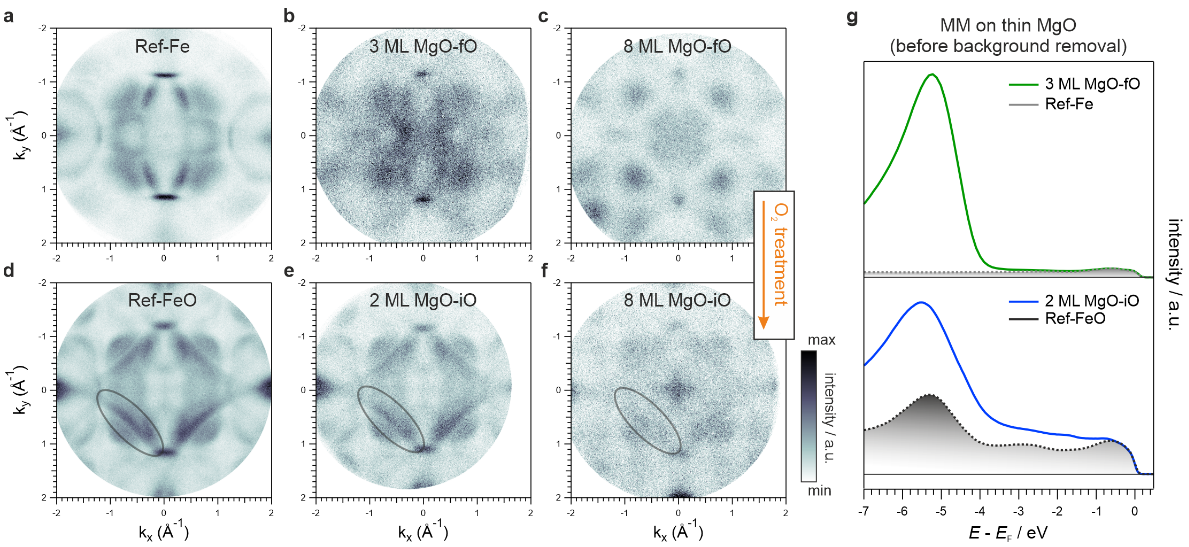

Figure S17*: Collection of momentum maps near the Fermi energy recorded at 21.2 eV with p-polarized light.* ***a)*** *Pristine Ref-Fe,* ***b)*** *3 ML MgO-fO without an interlayer, and* ***c)*** *8 ML MgO-fO without an interlayer. The work function evolves from 4.1 eV for Ref-Fe to 2.4 eV for 3 ML MgO-fO and increases slightly to 2.8 eV for the thick MgO-fO film in c).* ***d)–f)*** *Momentum maps corresponding to* ***d)*** *Ref-FeO,* ***e)*** *2 ML MgO-iO, and* ***f)*** *an 8 ML MgO-iO film obtained through subsequent oxygen treatment. The work function decreases across these samples from 4.4 eV to 4.0 eV to 3.6 eV, with each sample containing an oxygen interlayer. A feature previously attributed to Fe-O interface states is highlighted in the bottom row of maps d)–f). The presence of this feature is associated with the presence of interfacial oxygen. To enhance statistics for thicker MgO films while ensuring comparability, all the maps were integrated over a small energy region of ±150 meV near E_F.* ***g)*** *EDCs of the thin MgO films before background removal.*

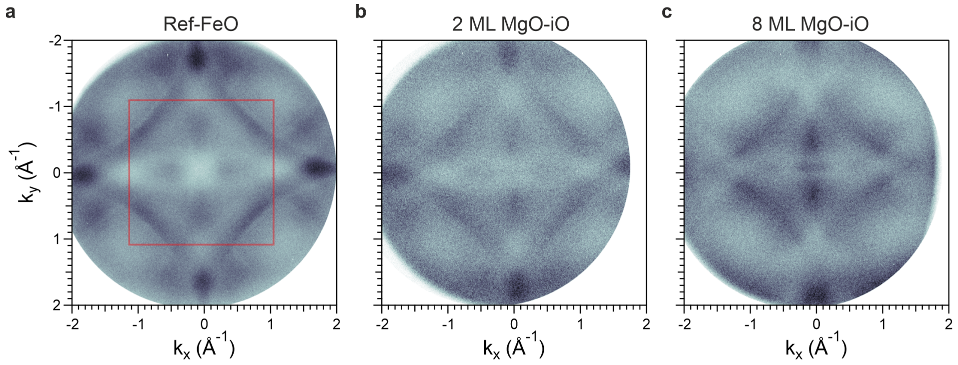

Figure S18*: Momentum maps recorded at -1.8 eV below E_F, where new electronic states for the samples with oxygen interlayer are observed. The maps belong to the following systems:* ***a)*** *Ref-FeO,* ***b)*** *2 ML MgO-iO, and* ***c)*** *8 ML MgO-iO. The strong agreement between the three maps indicates a shared origin for the observed peak in the EDC and a periodicity that matches the SBZ of the Fe(100) substrate. Measurements were taken at 21.2 eV using p-polarized light.*

Supporting References:

1. Oh, H., Lee, S. B., Seo, J., Min, H. G. & Kim, J.-S. Chemical structure of the interface between MgO films and Fe(001). *Appl. Phys. Lett.* **82**, 361–363 (2003).

2. Rossi, G., Sirotti, F., Cherepkov, N. A., Combet Farnoux, F. & Panaccione, G. 3p fine structure of ferromagnetic Fe and Co from photoemission with linearly polarized light. *Solid State Commun.* **90**, 557–562 (1994).

3. Meyerheim, H. L. *et al.* Geometrical and Compositional Structure at Metal-Oxide Interfaces: MgO on Fe(001). *Phys. Rev. Lett.* **87**, 076102 (2001).

4. Tait, S. L. *et al.* Metal−Organic Coordination Interactions in Fe−Terephthalic Acid Networks on Cu(100). *J. Am. Chem. Soc.* **130**, 2108–2113 (2008).

5. Corneille, J. S., He, J. & Goodman, D. W. XPS characterization of ultra-thin MgO films on a Mo(100) surface. *Surf. Sci.* **306**, 269–278 (1994).

6. Nelin, C. J. *et al.* Surface core-level binding energy shifts for MgO(100). *Phys. Chem. Chem. Phys.* **16**, 21953–21956 (2014).

7. Müller, M., Matthes, F. & Schneider, C. M. Photoemission study of the Fe(001)/MgO interface for varying oxidation conditions of magnesium oxide. *J. Appl. Phys.* **101**, 09G519 (2007).

8. Handschak, D. *et al.* Structural investigation of the three-layer system MgO/Fe/GaAs(001) by means of photoelectron spectroscopy and diffraction. *Phys. Rev. B* **88**, 045313 (2013).

9. Sinković, B., Johnson, P. D., Brookes, N. B., Clarke, A. & Smith, N. V. Magnetic structure of oxidized Fe(001). *Phys. Rev. Lett.* **65**, 1647 (1990).

10. Schmid, M., Steinrück, H. & Gottfried, J. M. A new asymmetric Pseudo-Voigt function for more efficient fitting of XPS lines. *Surf. Interface Anal.* **46**, 505–511 (2014).

11. Seah, M. P. The quantitative analysis of surfaces by XPS: A review. *Surf. Interface Anal.* **2**, 222–239 (1980).

12. Childs, K. D. *et al. Handbook of Auger Electron Spectroscopy: A Book of Reference Data for Identification and Interpretation in Auger Electron Spectroscopy*. (Physical Electronics, 1995).

13. Shinotsuka, H., Tanuma, S., Powell, C. J. & Penn, D. R. Calculations of electron inelastic mean free paths. XII. Data for 42 inorganic compounds over the 50 eV to 200 keV range with the full Penn algorithm. *Surf. Interface Anal.* **51**, 427–457 (2019).
